# When Weight Matters: How Obesity Impacts Reproductive Health and Pregnancy-A Systematic Review

**DOI:** 10.1007/s13679-025-00629-9

**Published:** 2025-04-16

**Authors:** Konstantina Barbouni, Vaidas Jotautis, Dimitra Metallinou, Athina Diamanti, Eirini Orovou, Alina Liepinaitienė, Petros Nikolaidis, Grigorios Karampas, Antigoni Sarantaki

**Affiliations:** 1 Midwifery Department, Faculty of Health & Care Sciences, University of West Attica, 12243 Egaleo, Athens Greece; 2 Faculty of Medicine, Kauno Kolegija Higher Education Institution, Pramonės Av. 20, 50468 Kaunas, Lithuania; 3 Midwifery Department, University of Western Macedonia, 50200 Ptolemaida, Greece; 4 Faculty of Natural Sciences, Department of Environmental Sciences, Vytautas Magnus University, Kaunas, Lithuania; 5 Embryoiatriki-Genetiki Ltd, 49 Kifisias Ave, Athens, Greece; 6 2nd Department of Obstetrics and Gynecology, Aretaieio University Hospital, Medical School, National and Kapodistrian University of Athens, Athens, Greece; 7 Department of Obstetrics and Gynecology, Scänes University Hospital, 21428 Malmö-Lund, Sweden

**Keywords:** Obesity, Reproductive Health, Assisted Reproductive Technology, Pregnancy Complications, Sperm, Epigenetics, Prolonged Time-to-Pregnancy

## Abstract

**Purpose of Review:**

This systematic review evaluates the impact of obesity on both male and female reproductive health, assisted reproductive technology (ART) outcomes, and pregnancy-related complications, providing a comprehensive synthesis of the evidence.

**Recent Findings:**

Obesity is a critical factor adversely affecting reproductive health, ART success rates, and pregnancy outcomes. Recent studies indicate hormonal disruptions, metabolic syndrome, and epigenetic modifications as central mechanisms linking obesity to infertility and adverse pregnancy results.

**Summary:**

A systematic search adhering to the Preferred Reporting Items for Systematic Reviews and Meta-Analyses (PRISMA) guidelines included 35 studies, focusing on obesity-related reproductive outcomes. The review highlights that obesity disrupts hormonal balance, including reductions in sex hormone-binding globulin (SHBG) and testosterone levels, alongside increased insulin resistance and chronic inflammation. These mechanisms impair ovarian function, endometrial receptivity, and sperm quality, resulting in prolonged time-to-pregnancy (TTP), reduced ART success rates, and increased miscarriage risk. During pregnancy, maternal obesity elevates risks of gestational diabetes mellitus (GDM), preeclampsia, and cesarean delivery while contributing to neonatal complications, such as macrosomia and neonatal intensive care unit (NICU) admissions. The findings emphasize the dual impact of maternal and paternal obesity on offspring health, particularly through epigenetic modifications leading to intergenerational metabolic dysfunction. This review underscores the necessity of preconception weight management, individualized ART protocols, and tailored antenatal care to mitigate obesity’s adverse effects on reproductive outcomes. Future research should focus on understanding male infertility mechanisms, optimizing ART interventions for individuals with obesity, and conducting longitudinal studies on the intergenerational impacts of obesity on reproductive health. This synthesis provides actionable insights to guide clinical practices and future investigations.

**Supplementary Information:**

The online version contains supplementary material available at 10.1007/s13679-025-00629-9.

## Introduction

Obesity, a global epidemic, is a complex condition characterized by excessive adiposity that significantly impacts health outcomes [1]. According to the World Health Organization (WHO), obesity prevalence has nearly tripled since 1975, with over 650 million adults worldwide classified as people with obesity in 2016 [2]. The implications of this growing health crisis extend beyond the well-established associations with cardiovascular disease, diabetes, and cancer, reaching into the domains of reproductive health and pregnancy [3–6]. For women of reproductive age, obesity poses unique challenges that influence fertility, pregnancy outcomes, and long-term maternal and child health [7].

Reproductive health is intricately linked to metabolic homeostasis, and obesity disrupts this balance through a range of mechanisms, including hormonal dysregulation, chronic inflammation, and altered energy metabolism. These disruptions can impair ovulatory function, reduce oocyte quality, and compromise endometrial receptivity, thereby diminishing fertility [8]. Obesity also interacts with assisted reproductive technologies (ART), often reducing their success rates and complicating treatment protocols [9].

While the reproductive consequences of obesity in women have been extensively studied due to their direct role in conception and pregnancy, emerging evidence underscores the importance of considering male factors as well. Male obesity contributes to subfertility through hormonal imbalances, impaired spermatogenesis, and epigenetic alterations that can affect embryonic development and offspring health. Obesity in men is frequently associated with hypogonadism, characterized by reduced testosterone levels and elevated estradiol due to increased aromatization in adipose tissue. These hormonal disturbances can impair spermatogenesis and reduce sperm motility, concentration, and morphology, thereby contributing to subfertility. Therefore, a dual-gender perspective is essential to fully understand the multifaceted impact of obesity on reproductive outcomes and to inform more holistic preconception and fertility care strategies [10].

The challenges posed by obesity extend beyond conception. During pregnancy, maternal obesity increases the risk of complications such as gestational diabetes mellitus (GDM), preeclampsia, preterm birth, and cesarean delivery [11]. Fetal and neonatal outcomes are also affected, with increased risks of macrosomia, congenital anomalies, and childhood obesity. These outcomes underscore the intergenerational impact of obesity, perpetuating cycles of adverse health outcomes [11].

Despite growing evidence, gaps remain in understanding the interactions between obesity, reproductive health, and pregnancy. Research often overlooks the mechanisms linking these factors and their clinical implications. Societal and healthcare barriers further hinder effective prevention and management. This systematic review synthesizes current evidence, exploring physiological mechanisms, clinical outcomes, and interventions. The findings aim to guide clinicians, researchers, and policymakers in addressing obesity's impact and improving reproductive and maternal-child health outcomes.

## Materials and Methods

### Study Design

This systematic review was conducted following the Preferred Reporting Items for Systematic Reviews and Meta-Analyses (PRISMA) guidelines [12]. This systematic review has been registered in the International Prospective Register of Systematic Reviews (PROSPERO) with ID number 631395.

### Research Question and Objectives

The primary objective of this review was to synthesize the existing literature on the impact of obesity on reproductive health and pregnancy outcomes. The specific aims included: a) Investigating the physiological and metabolic mechanisms linking obesity to reproductive dysfunction; b) Exploring the impact of obesity on male fertility; c) Analyzing the effects of obesity on ART outcomes and d) Evaluating maternal and neonatal outcomes associated with obesity during pregnancy.

### Data Sources and Search Strategy

A comprehensive search was conducted in the following electronic databases from 2004 to 2024: PubMed/MEDLINE; Embase; Cochrane Library and Web of Science.

The search strategy included Medical Subject Headings (MeSH), and free-text terms related to obesity ("obesity","body mass index","BMI"), reproductive health ("fertility","assisted reproductive technologies","IVF"), and pregnancy outcomes ("gestational diabetes","preeclampsia","neonatal outcomes"). Boolean operators (AND, OR) and database-specific filters were applied to refine the search.

### Inclusion and Exclusion Criteria

To determine the eligibility of studies, the Population-Intervention- Comparison-Outcome-Studies (PICOS) framework was used: a) Population: Studies involving individuals of reproductive age (18–45 years) with obesity [Body Mass Index (BMI) ≥ 30 kg/m^2^]; b) Interventions/Exposures: Studies addressing obesity and its impact on fertility, ART outcomes, and pregnancy-related outcomes; c) Comparators: Normal-weight individuals (BMI 18.5–24.9 kg/m^2^) or other BMI categories; d) Outcomes: Fertility metrics (e.g., ovulation, oocyte quality, sperm quality), ART success rates, maternal complications (e.g., gestational diabetes, preeclampsia), and neonatal outcomes (e.g., birth weight, preterm birth); e) Study Design: Observational studies (cohort, case–control, cross-sectional) and randomized controlled trials (RCTs).

Exclusion criteria were: a) studies focusing solely on populations outside reproductive age; b) non-human studies or animal models; c) articles lacking primary data (e.g., opinion pieces, narrative reviews) and d) studies without BMI stratification or insufficient obesity-related data.

### PRISMA Process

During the identification phase, a total of 880 records were identified, comprising 850 records from database searches (PubMed, Embase, Cochrane Library, and Web of Science) and an additional 30 records from other sources, such as references and grey literature.

In the screening phase, 230 duplicate records were removed, leaving 650 records for further review. Following title and abstract screening, 490 records were excluded for being non-relevant or outside the scope of the review. Consequently, 160 full-text articles were assessed for eligibility.

In the eligibility phase, 110 full-text articles were excluded for various reasons, including 45 studies lacking BMI stratification, 30 studies with inadequate data on reproductive health or pregnancy outcomes, and 35 studies that did not meet the inclusion criteria based on study design. Ultimately, 50 full-text articles were deemed eligible.

During the inclusion phase, 15 studies were excluded after quality assessment due to high risk of bias, such as poor comparability or inadequate outcome assessment. This process resulted in a final inclusion of 35 studies in the systematic review.

**Fig. 1:**
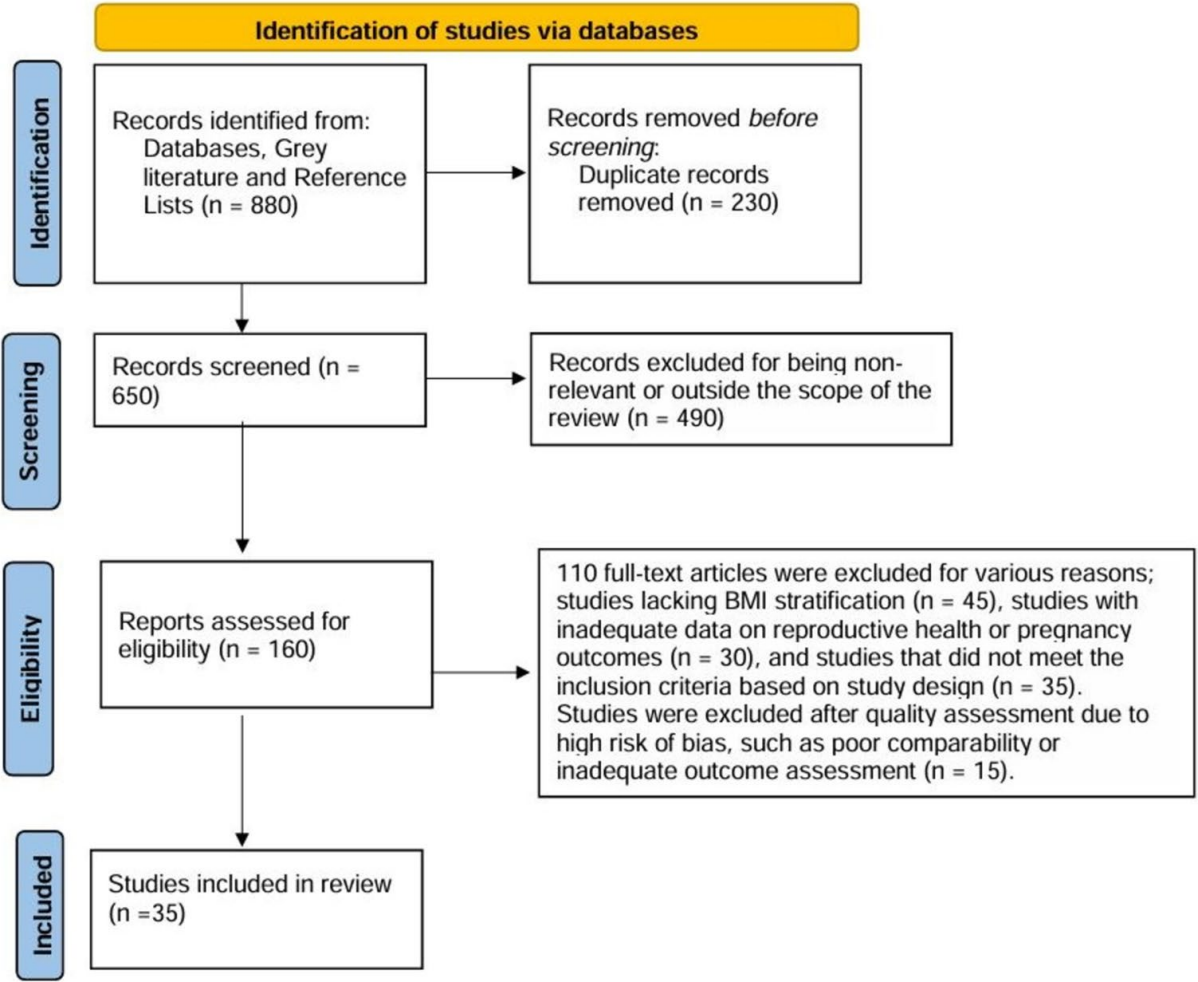
Flowchart for the study selection process

The flowchart of the selection process is illustrated in Fig. 1.

### Data Extraction

A standardized data extraction form was developed to collect the following information: a) Study Characteristics: Authors, year, country, study design, sample size; b) Population Details: Age, BMI categories, baseline characteristics; c) Outcomes Assessed: Fertility metrics, ART success rates, maternal and neonatal outcomes and d) Key Findings and Conclusions: Summary of results and main conclusions.

Data extraction was performed independently by two reviewers. Any disagreements were resolved through consensus.

### Quality Assessment

The quality and risk of bias of included studies were evaluated using the Newcastle–Ottawa Scale (NOS) [13] for observational studies and the Risk of Bias Assessment 2 Tool (RoB 2 Tool) [14] for RCTs. Studies were scored on the selection of participants, comparability of groups, and outcome assessment. Any disagreements between reviewers regarding quality or risk of bias assessments were resolved through discussion and consensus, with involvement of a third reviewer when necessary. The quality assessment is provided in Supplementary File 1.

### Data Synthesis

Findings from the included studies were summarized narratively, categorized into themes: a) Mechanisms linking obesity to reproductive dysfunction; b) Impact of obesity on male fertility; c) Effects of obesity on ART outcomes, and d) Maternal and neonatal outcomes associated with obesity during pregnancy.

## Results

The systematic review included a total of 35 studies [15–49]. The included studies represented a wide geographical distribution, with research conducted across North America, Europe, Asia, and Oceania. Notable contributions came from the USA, UK, China, Australia, and several European countries, reflecting the global concern over obesity-related reproductive health issues and adding cultural and clinical diversity to the findings. The study designs predominantly consisted of retrospective and prospective cohort studies, along with some cross-sectional studies and analyses of clinical trial data. Sample sizes varied widely, ranging from small cohorts of fewer than 50 participants to large population-based analyses with over 10,000 subjects. The studies covered diverse populations, including women undergoing ART like in vitro fertilization (IVF), pregnant women stratified by BMI, and men evaluated for fertility. They measured parameters such as gestational and neonatal outcomes, hormonal profiles, ovarian and sperm morphology, embryo quality, and live birth rates.

Summary of the included studies’ characteristics is displayed in Tables 1, 2, 3, and 4.

**Table 1:** Studies regarding mechanisms linking obesity to reproductive dysfunction

Ν	Authors	Year	Country	Type of Study	Study Sample & Comparators	Investigations	Outcomes	Main Conclusions
1	Grieger et al. [15]	2019	Multicenter (Australia, Ireland, New Zealand, UK)	Retrospective cohort study	5,519 low-risk nulliparous women with and without metabolic syndrome (MetS); stratified by BMI (< 30 vs ≥ 30 kg/m^2^)	TTP, infertility, and individual MetS components	MetS was associated with longer TTP and a 62% greater risk of infertility, independent of obesity. Raised triglycerides and reduced HDL-C were strongly associated with infertility	MetS, regardless of obesity, prolongs TTP and increases infertility risk. Individual metabolic abnormalities (e.g., HDL-C, triglycerides) play significant roles in delayed fertility
2	Lin et al. [16]	2017	China	Observational study	189 infertile women undergoing IVF; grouped by BMI (normal: 18.5–23.9 kg/m^2^, overweight/obese: ≥ 24 kg/m^2^)	Good-quality embryo rate, leptin levels in serum and follicular fluid, IVF outcomes (e.g., clinical pregnancy rate, live birth rate)	Overweight women/women with obesity showed reduced good-quality embryo rate and lower clinical pregnancy and live birth rates. Elevated follicular fluid leptin levels were negatively correlated with embryo quality and fertility outcomes	High leptin levels in overweight women/women with obesity impair granulosa cell function and embryo quality, linking obesity to poor fertility outcomes
3	Kazemi et al. [17]	2020	USA	Multicenter cross-sectional study	111 reproductive-aged women (18–45 years); evaluated dietary quality (aMED, DASH scores) and ovarian morphology (FNPO, OV)	Diet quality indices, ovarian morphology on ultrasonography, metabolic (BMI, IR, hyperandrogenism) and hormonal markers	Higher adherence to aMED and DASH scores was associated with decreased ovarian volume (OV) and follicle number (FNPO) through reductions in obesity (BMI/WC), insulin resistance (glucose AUC), and hyperandrogenism (FAI)	Diet quality indirectly improves ovarian morphology via metabolic and hormonal mediators. Mediterranean and DASH diets may benefit women with ovarian dysfunction, emphasizing dietary interventions
4	Oldfield et al. [18]	2023	USA	Prospective longitudinal study	42 women (21 with obesity [≥ 35% body fat], 21 without obesity [< 35% body fat]), aged 19–38 years, with regular menstrual cycles	Antral follicle dynamics (recruitment, dominance, selection), endocrine hormones (AMH, LH, FSH, estradiol, progesterone), luteal phase defects	Women with obesity displayed fewer recruitment events (P = 0.010), lower selectable follicles (P = 0.022), reduced AMH levels (P = 0.023), and increased luteal phase defects (76% vs. 29%, P = 0.002). Ovulatory follicles were selected at smaller diameters in women with obesity (P = 0.001)	Obesity is associated with suppressed ovarian antral follicle dynamics and altered endocrine function despite regular menstrual cycles, which may underlie suboptimal reproductive outcomes

aMED—Alternate Mediterranean Diet Score; AMH—Anti-Müllerian Hormone; AUC—Area Under the Curve (glucose tolerance/insulin resistance evaluation); BMI—Body Mass Index; DASH—Dietary Approaches to Stop Hypertension (dietary score); FAI—Free Androgen Index; FNPO—Follicle Number Per Ovary; FSH—Follicle Stimulating Hormone; HDL-C—High-Density Lipoprotein Cholesterol; IR—Insulin Resistance; LH—Luteinizing Hormone; MetS—Metabolic Syndrome; OV—Ovarian Volume; TTP—Time to Pregnancy; WC—Waist Circumference.

**Table 2:** Studies regarding impact of obesity on male fertility

Ν	Authors	Year	Country	Type of Study	Study Sample & Comparators	Investigations	Outcomes	Main Conclusions
1	Bieniek et al. [19]	2016	USA	Retrospective cohort study	1,068 men seeking infertility evaluation; stratified by BMI (normal weight, overweight, obese)	Reproductive hormones (total testosterone, free testosterone, estradiol, SHBG) and semen parameters (volume, count, motility, morphology)	Men with obesity had significantly lower total testosterone, free testosterone, and SHBG levels compared to normal-weight men. Estradiol levels were higher in men with obesity. No significant differences in semen parameters were observed across BMI categories	Obesity in men is associated with hypogonadism (lower testosterone and SHBG) and elevated estradiol levels, but it does not significantly affect semen parameters
2	Kort et al. [20]	2006	USA	Retrospective cohort study	520 men undergoing routine semen analysis; grouped by BMI (normal: 20–24 kg/m^2^, overweight: 25–30 kg/m^2^, obese: > 30 kg/m^2^)	Semen parameters (concentration, motility, morphology) and sperm DNA integrity (DFI)	Obese men had significantly fewer normal-motile sperm (0.7 × 10⁶) compared to overweight (3.6 × 10⁶) and normal BMI men (18.6 × 10⁶). Sperm DFI increased with BMI (normal: 19.9%, overweight: 25.8%, obese: 27.0%)	Higher BMI is associated with poorer sperm quality, including reduced motility and increased DNA fragmentation. Weight reduction may improve fertility potential
3	La Vignera et al. [21]	2012	Italy	Prospective observational study	150 healthy non-smoking men stratified by BMI: normal weight (n = 50), overweight (n = 50), obese (n = 50)	Conventional (density, motility, morphology) and non-conventional sperm parameters (mitochondrial function, apoptosis, chromatin integrity, DNA fragmentation)	Overweight and men with obesity had lower sperm motility and normal morphology compared to controls. Men with obesity showed increased sperm apoptosis, chromatin decondensation, and DNA fragmentation	Increased BMI negatively impacts sperm quality, emphasizing the need for weight management programs in addressing male infertility
4	Martini et al [22]	2010	Argentina	Prospective observational study	794 men stratified by BMI: normal weight (18.5–24.9), overweight (25–29.9), obese (≥ 30)	Semen quality parameters (motility, morphology, viability, nuclear maturity, etc.), functional markers (NAG, fructose, citric acid), T levels	BMI was negatively associated with total and rapid sperm motility and NAG levels. Positive association was observed with seminal fructose levels. No significant changes in sperm morphology, concentration, or T levels were noted	Obesity negatively affects seminal motility and epididymal function (NAG secretion), potentially impacting male fertility. No significant alterations in testosterone levels or sperm morphology were observed
5	Paasch et al. [23]	2010	Germany	Case-cohort study	2,157 males aged 17–67 years; stratified by BMI (underweight, normal weight, overweight, obese)	Semen parameters (TSC, TCN, motility), serum hormones (T, FSH, LH, inhibin-B), CASA sperm motion analysis	BMI negatively correlated with TSC (r = − 0.07, P = 0.0005), TCN (r = − 0.13, P = 0.000001), T (− 0.15, P = 0.000001), and inhibin-B (− 0.07, P = 0.003). In men aged 20–30, BMI significantly impacted TSC (r = − 0.102, P < 0.05)	Both BMI and age influence male fertility, with age having a stronger overall impact. High BMI is particularly detrimental to TSC and TCN in younger men (20–30 years)
6	Raad et al. [24]	2019	Lebanon	Prospective cohort study	128 infertile men stratified by BMI: lean (< 25 kg/m^2^), overweight (25–29.9 kg/m^2^), obese (≥ 30 kg/m^2^)	Sperm motility, chromatin integrity, DNA methylation, oxidative stress, embryo morphokinetics	Men with obesity showed increased sperm chromatin damage (TB + cells, p < 0.01), lower DNA methylation (5-mC, p < 0.05), higher oxidative stress (p < 0.001), and delayed embryonic cell cycles (CC1, CC3, p < 0.05)	Paternal obesity negatively affects sperm molecular characteristics and early embryo development. This highlights the importance of paternal weight management in fertility treatments

5-mC—5-methylcytosine (DNA methylation marker); BMI—Body Mass Index; CASA—Computer-Assisted Sperm Analysis; CC1—Cell Cycle 1 (embryonic cell cycle phase); CC3—Cell Cycle 3 (embryonic cell cycle phase); DFI—DNA Fragmentation Index; FSH—Follicle Stimulating Hormone; LH—Luteinizing Hormone; NAG—Neutral Alpha-Glucosidase (epididymal function marker); SHBG—Sex Hormone Binding Globulin; T – Testosterone; TB +—Terminal deoxynucleotidyl transferase dUTP Nick-End Labeling Positive (indicator of sperm DNA fragmentation); TCN—Total Cell Number; TSC—Total Sperm Count.

**Table 3:** Studies regarding impact of obesity on ART outcomes

Ν	Authors	Year	Country	Type of Study	Study Sample & Comparators	Investigations	Outcomes	Main Conclusions
1	Pinborg et al. [25]	2011	Denmark	Longitudinal multicenter cohort study	487 infertile women undergoing IVF/ICSI; BMI categories: underweight (< 18.5), normal weight (18.5–24.9), overweight (25–29.9), obese (≥ 30 kg/m^2^)	IVF outcomes: gonadotropin dose, oocyte retrieval, embryo development, pregnancy, live birth	Women with obesity required higher gonadotropin doses, had fewer retrieved oocytes (8.3 vs. 9.9), and fewer embryos (4.7 vs. 6.2). Live-birth rates per cycle were lower in women with obesity (15.2%) compared to normal-weight women (21.5%)	Female obesity negatively impacts IVF outcomes, including lower oocyte retrieval and live-birth rates. BMI is a significant independent predictor of IVF success
2	Devranoğlu et al. [26]	2024	Turkey	Retrospective cross-sectional study	833 IVF/ICSI cycles among women with diminished ovarian reserve (DOR); stratified by BMI (non-obese: < 30 kg/m^2^ vs obese: ≥ 30 kg/m^2^)	Gonadotropin dose, stimulation duration, number of MII oocytes, and clinical pregnancy rates	Obese patients required higher gonadotropin doses and longer stimulation durations, with fewer MII oocytes retrieved compared to non-obese patients. No significant differences in clinical pregnancy rates between groups	Obesity negatively impacts ovarian response to stimulation in DOR patients, but clinical pregnancy rates are unaffected. Highlights need for personalized protocols in DOR patients with obesity undergoing ART
3	Caillon et al. [27]	2015	France	Retrospective analysis	558 women undergoing IVF, stratified by BMI (normal weight, overweight, obese)	IVF cycle outcomes, ovarian stimulation response, embryo quality, pregnancy outcomes	Higher gonadotropin doses required for ovarian stimulation in women with obesity. Increased transfer cancellation and miscarriage rates noted in women with obesity	Obesity does not compromise IVF outcomes when stimulation protocols are adjusted. However, women with obesity have poorer live birth rates and higher miscarriage risks, suggesting metabolic influences on reproductive outcomes
4	Haghighi et al. [28]	2012	Iran	Retrospective cohort study	230 women undergoing first IVF cycle; grouped by BMI (< 20, 20–27.9, ≥ 28 kg/m^2^)	IVF outcomes: number of aspirated follicles, mature oocytes, embryos transferred, endometrial thickness, clinical pregnancy rate	BMI had no significant effect on IVF outcomes (e.g., follicles, oocytes, or pregnancy rates). A significant difference in endometrial thickness was observed among BMI groups	BMI does not adversely affect IVF outcomes, but weight loss in women with obesity is advised to reduce obstetric complications during pregnancy
5	Leary et al. [29]	2015	UK	Retrospective observational study	218 oocytes from 29 women (BMI ≥ 25 vs BMI < 25 kg/m^2^) undergoing IVF/ICSI; metabolic activity measured in 150 embryos from 29 additional women	Oocyte morphology, developmental kinetics, metabolic activity (glucose, pyruvate, amino acids, triglycerides), and blastocyst cell counts	Oocytes from women with obesity were smaller and less likely to develop into blastocysts. Blastocysts exhibited faster morula-stage progression, fewer trophectoderm cells, reduced glucose uptake, altered amino acid metabolism, and higher triglyceride levels	Maternal obesity is linked to impaired oocyte quality and abnormal embryo metabolism, which may impact implantation, fetal development, and long-term offspring health
6	Bellver et al. [30]	2013	Spain	Retrospective cohort study	9,587 first ovum donation cycles; recipients stratified by BMI (lean: < 20 kg/m^2^, normal: 20–24.9 kg/m^2^, overweight: 25–29.9 kg/m^2^, obese: ≥ 30 kg/m^2^)	Implantation, clinical pregnancy, live birth, and miscarriage rates	As BMI increased, implantation (40.4% to 30.9%), clinical pregnancy (56.9% to 45.3%), and live birth rates (38.6% to 27.7%) decreased significantly. No differences in clinical miscarriage rates were observed across BMI groups	Obesity in recipients of ovum donation negatively impacts uterine receptivity, reducing implantation, pregnancy, and live birth rates. Preconception weight management is recommended to improve outcomes
7	Zhang et al. [31]	2015	USA	Retrospective cohort study	439 participants undergoing IVF; grouped by BMI (normal, overweight, obese) and IVF protocol (conventional vs minimal stimulation)	Oocyte number, maturity (MII), fertilization, embryo development, pregnancy, and live birth rates	In conventional IVF, women with obesity had significantly fewer total and mature (MII) oocytes compared to normal BMI women. No such differences were observed in minimal stimulation IVF. Pregnancy and live birth rates were not significantly different between BMI groups in either protocol	Minimal stimulation IVF may yield healthier oocytes and better outcomes in women with obesity compared to conventional IVF. Obesity negatively impacts outcomes in conventional IVF
8	Luke et al. [32]	2011	USA	Multicenter retrospective study	45,163 ART embryo transfers; stratified by BMI and oocyte source (autologous vs donor)	Failure to achieve clinical pregnancy and live birth rates	Increasing BMI was associated with higher failure rates to achieve clinical pregnancy and live birth using autologous oocytes but not donor oocytes	Obesity adversely affects ART outcomes with autologous oocytes, emphasizing the importance of preconception weight management to improve ART success
9	Veleva et al. [33]	2008	Finland	Retrospective cohort study	1,000 women undergoing IVF; stratified by BMI (normal: 18.5–24.9 kg/m^2^, overweight: 25–29.9 kg/m^2^, obese: ≥ 30 kg/m^2^)	IVF outcomes (clinical pregnancy, live birth rates, miscarriage rates)	Women with obesity had significantly lower clinical pregnancy and live birth rates and higher miscarriage rates compared to normal-weight women	Obesity is associated with poorer IVF outcomes, highlighting the need for preconception weight management in women with obesity undergoing ART
10	Chavarro et al. [34]	2012	USA	Prospective cohort study	170 women undergoing 233 ART cycles; BMI groups (< 20, 20–22.4, 22.5–24.9, 25–29.9, ≥ 30 kg/m^2^)	ART outcomes: MII oocyte yield, peak estradiol levels, endometrial thickness, fertilization, embryo quality, clinical pregnancy, and live birth rates	Higher BMI was associated with lower peak estradiol levels, MII oocyte yield, and live birth rates. Short-term weight loss was linked to improved MII yield but did not affect clinical outcomes	BMI negatively impacts ART outcomes, including live birth rates. Short-term weight loss may improve oocyte quality but has limited impact on clinical pregnancy or live birth rates
11	Kim et al. [35]	2020	USA	Prospective cohort study	2,013 couples undergoing IVF; BMI and %BF evaluated separately in males and females	Ovarian reserve (FSH, AMH, AFC), oocyte yield, TMSC, sperm concentration	Obesity by BMI and %BF was associated with lower AMH and higher AFC in women. No differences in mature oocyte yield or sperm concentration across adiposity groups. Males with high %BF had reduced TMSC	Obesity, especially %BF, impacts ovarian reserve and TMSC but does not significantly limit gamete production for ART. %BF may be more accurate than BMI for assessing obesity-related fertility risks
12	He et al. [36]	2019	China	Secondary analysis of an RCT	1,508 women with PCOS undergoing first IVF cycle; stratified by MetS status (MetS vs non-MetS)	IVF stimulation, fertility, cumulative live birth rates, metabolic factors	Women with MetS had poorer ovarian response, fewer retrieved and available embryos, and lower cumulative live birth rates. Higher gonadotropin doses and longer stimulation were required in the MetS group	MetS negatively impacts fertility and IVF outcomes in PCOS women, emphasizing the need for preconception metabolic evaluation and management
13	Kudesia et al. [37]	2018	USA	Retrospective cohort study	51,198 women undergoing first autologous IVF cycle (BMI: underweight, normal, overweight, obese class-I, obese class-II/III)	Cycle cancellation, number of oocytes retrieved, usable embryos, ongoing clinical pregnancy (OCP)	Overweight and obese women had higher odds of cycle cancellation (aOR: 1.17–1.50), fewer oocytes retrieved (aIRR: 0.93 for obese class-II/III), and lower OCP rates (aOR: 0.89–0.86). Negative effects were most pronounced in obese class-II/III, women with PCOS, or ovulatory dysfunction	BMI above the normal range is an independent negative factor for IVF outcomes. Weight management is recommended, particularly for women with obesity with PCOS or ovulatory dysfunction, to improve ART success
14	Orvieto et al. [38]	2020	Israel	Cohort prospective study	189 women undergoing ovarian stimulation (157 nonobese, 32 obese; follicles > 15 mm)	Oocyte recovery rate, fertilization (2PN/oocyte), top-quality embryos (TQE/zygote)	No significant differences between obese and nonobese patients in oocyte recovery rate, fertilization, or TQE rates from follicles > 15 mm	Patients with obesity yield competent oocytes and embryos comparable to nonobese patients when follicles > 15 mm are aspirated. Suggests that poor IVF outcomes in patients with obesity are more related to poor response to stimulation
15	Legge et al. [39]	2014	Canada	Retrospective cohort study	752 women undergoing 951 IVF/IVF-ICSI cycles; BMI groups: normal weight (< 25 kg/m^2^), overweight (25–29.9 kg/m^2^), obese (≥ 30 kg/m^2^)	Total gonadotropin dose, cycle cancellation, clinical pregnancy, live birth rates	No significant differences between BMI groups in gonadotropin dose requirements, cycle cancellation rates, clinical pregnancy rates, or live birth rates. Women with obesity had longer infertility duration and higher prevalence of PCOS	At the studied center, BMI did not significantly impact IVF clinical outcomes, though other studies report conflicting results. Weight management remains essential for overall reproductive health

%BF—Percent Body Fat; 2PN—Two Pronuclei (fertilized oocytes); AFC—Antral Follicle Count; AMH—Anti-Müllerian Hormone; aIRR—Adjusted Incidence Rate Ratio; aOR—Adjusted Odds Ratio; ART—Assisted Reproductive Technology; BMI—Body Mass Index; DOR—Diminished Ovarian Reserve; FSH—Follicle Stimulating Hormone; ICSI—Intracytoplasmic Sperm Injection; IVF—In Vitro Fertilization; MetS—Metabolic Syndrome; MII—Metaphase II (Mature Oocytes); OCP—Ongoing Clinical Pregnancy; PCOS—Polycystic Ovary Syndrome; RCT—Randomized Controlled Trial; TMSC—Total Motile Sperm Count; TQE—Top-Quality Embryos.

**Table 4:** Studies regarding maternal and neonatal outcomes associated with obesity during pregnancy

Ν	Authors	Year	Country	Type of Study	Study Sample & Comparators	Investigations	Outcomes	Main Conclusions
1	Aly et al. [40]	2009	USA	Retrospective analysis	14,183 singleton mother-infant pairs; stratified by maternal BMI (non-obese, obese, morbidly obese)	Gestational age, birth weight, complications (e.g., anemia, hypertension, diabetes), cesarean rates	Obesity and morbid obesity were associated with more complications (e.g., hypertension, diabetes) and higher cesarean delivery rates, but not directly with prematurity after adjusting for confounders	Prematurity is linked to medical complications associated with obesity rather than obesity itself. Enhanced prenatal care to manage associated conditions may improve outcomes
2	Joy et al. [41]	2008	USA	Retrospective cohort study	12,915 singleton term pregnancies; stratified by maternal BMI (normal: 20–24.9, obese: ≥ 30, subgroups by severity)	Maternal morbidities (e.g., gestational hypertension, diabetes), neonatal outcomes (e.g., large-for-gestational-age infants)	Obesity increased risks for gestational hypertension, diabetes, cesarean deliveries, and large-for-gestational age infants. Risks increased with obesity severity	Maternal obesity, particularly severe obesity, significantly raises risks of adverse maternal and neonatal outcomes, requiring tailored prenatal care
3	Sabolović Rudman et al. [42]	2019	Croatia	Retrospective cohort study	51 pregnant women with obesity and 50 normal BMI pregnant women with GDM diagnosed by IADPSG criteria	Gestational weight gain, gestational hypertension, neonatal birth weight, fetal hypertrophy, gestational age, mode of delivery	Women with obesity had significantly higher gestational weight gain (15.27 vs. 10.56 kg), higher rates of hypertension (23.53% vs. 2%), and more hypertrophic newborns (21.57% vs. 4%). No significant differences in gestational age or mode of delivery were observed	Obesity exacerbates the adverse effects of GDM, including increased weight gain, hypertension, and fetal hypertrophy. Weight management and careful monitoring are essential for improving outcomes
4	Shen et al. [43]	2018	China	Prospective cohort study	2,292 pregnant women in Shanghai, BMI categories: underweight (< 18.5 kg/m^2^), normal weight (18.5–23.9 kg/m^2^), overweight (24–28 kg/m^2^), obese (> 28 kg/m^2^)	Gestational weight gain, delivery mode, maternal complications, neonatal outcomes (macrosomia, birth weight)	Pre-pregnancy obesity increased risks of excessive gestational weight gain (OR 3.58), macrosomia (OR 2.24), cesarean delivery (OR 2.04), and maternal complications (OR 1.53). No significant differences in preterm birth or neonatal mortality were observed	Pre-pregnancy obesity significantly impacts maternal and neonatal outcomes. Interventions targeting maternal obesity are critical to improving pregnancy outcomes in urban populations
5	Magriples et al. [44]	2009	USA	Secondary analysis of RCT data	841 young pregnant women (ages 14–25), BMI groups (underweight, normal weight, overweight, obese)	Maternal weight gain, cesarean delivery, neonatal outcomes (SGA, LGA), birth weight	Obesity was associated with higher cesarean delivery rates (OR 2.30) and a shift toward LGA infants. Weight gain influenced SGA and LGA risks but not cesarean delivery. Overweight and women with obesity had higher cesarean delivery risks, regardless of infant size	Pre-pregnancy BMI is a stronger predictor of cesarean delivery risk than gestational weight gain. Weight management before and during pregnancy can reduce adverse outcomes
6	Melchor et al. [45]	2019	Spain	Historical cohort study	16,609 women with singleton pregnancies (2013–2017); BMI categories: normal weight (18.5–24.9 kg/m^2^) vs obese (≥ 30 kg/m^2^)	Maternal (preeclampsia, cesarean delivery, GBS colonization) and neonatal outcomes (NICU admission, macrosomia, neonatal mortality)	Obesity increased the risk of preeclampsia (aOR 2.20), cesarean section (aOR 2.76), and NICU admission (aOR 1.34). Macrosomia rates were higher in women with obesity (≥ 4000 g: aOR 2.09; ≥ 4500 g: aOR 3.09). No differences in preterm birth, stillbirth, or neonatal mortality were observed	Maternal obesity is a significant risk factor for adverse pregnancy and neonatal outcomes, emphasizing the need for high-risk management in this population
7	Dickey et al. [46]	2012	USA	Retrospective cohort study	46,164 pregnancies (27,242 singletons, 18,922 twins) following IVF; stratified by maternal BMI (< 18.5, 18.5–24.9, 25–29.9, 30–34.9, ≥ 35 kg/m^2^)	Maternal weight, BMI, preterm birth (< 37 weeks), very preterm birth (< 32 weeks), neonatal birth weight, NICU admission	Preterm birth and NICU admission rates were significantly higher in twin pregnancies compared to singletons across all BMI categories. In singleton pregnancies, higher maternal BMI reduced the risk of preterm birth but increased the risk of macrosomia. In twin pregnancies, obesity did not significantly impact preterm birth or neonatal outcomes	Maternal BMI influences preterm birth and neonatal outcomes differently in singleton and twin pregnancies. Obesity may reduce preterm birth risk in singletons but has limited impact on twin outcomes
8	Adwani et al. [47]	2021	Saudi Arabia	Cross-sectional retrospective study	186 pregnant women with obesity (class I-III obesity); data collected from King Khaled Hospital	Maternal and neonatal outcomes: preeclampsia, episiotomy, perineal tears, preterm birth, APGAR scores, NICU admission, neonatal mortality	Obesity class III was associated with higher rates of episiotomy and perineal tears. No significant relationship was found between obesity classes and gestational diabetes, neonatal mortality, or NICU admission	Maternal obesity, particularly class III, impacts pregnancy and delivery outcomes, emphasizing the need for early antenatal care and obesity management
9	Bhandari et al. [48]	2015	UK	Retrospective observational study	414 women with recurrent miscarriage (RMC); grouped by BMI (normal, overweight, obese)	Pregnancy loss patterns, TTP intervals	Women with obesity with RMC were more likely to achieve pregnancy faster but with an increased risk of first-trimester miscarriages, particularly anembryonic pregnancies	Women with obesity with RMC exhibit"biological superfertility"but are at higher risk for early pregnancy losses, likely due to metabolic and endometrial dysfunctions
10	Yogev et al. [49]	2008	USA	Cohort study	4,830 patients with GDM stratified by BMI (obese: 30–35 kg/m^2^, morbidly obese: ≥ 35 kg/m^2^)	Pregnancy outcomes: LGA, macrosomia, cesarean delivery, NICU admission, metabolic complications	No significant differences in outcomes between obese and morbidly obese patients, but diet-treated patients had worse outcomes compared to insulin-treated patients in both groups	Pregnancy outcomes are compromised in women with obesity with GDM regardless of the level of obesity. Insulin treatment improves outcomes, especially in morbidly obese patients

APGAR—Appearance, Pulse, Grimace, Activity, Respiration (newborn evaluation score); aOR—Adjusted Odds Ratio; BMI—Body Mass Index; GDM—Gestational Diabetes Mellitus; GBS—Group B Streptococcus; IADPSG—International Association of the Diabetes and Pregnancy Study Groups; IVF—In Vitro Fertilization; LGA—Large for Gestational Age; NICU—Neonatal Intensive Care Unit; OR—Odds Ratio; RCT—Randomized Controlled Trial; RMC—Recurrent Miscarriage; SGA—Small for Gestational Age; TTP—Time to Pregnancy.

### Mechanisms Linking Obesity to Reproductive Dysfunction

Obesity significantly influences various mechanisms linked to reproductive dysfunction, including metabolic, hormonal, and ovarian morphological changes.

### Impact of Obesity-Driven Metabolic Dysregulation

Grieger et al. [15] identified that metabolic syndrome (MetS), which often co-occurs with obesity, is independently associated with longer time to pregnancy (TTP) and increased infertility risk. Key metabolic markers, such as raised triglycerides and reduced high-density lipoprotein cholesterol (HDL-C), were strongly correlated with infertility, highlighting the role of specific metabolic abnormalities beyond overall obesity in delaying fertility​.

### Hormonal Changes and Embryo Quality

Lin et al. [16] demonstrated that elevated leptin levels in the follicular fluid of overweight and women with obesity were negatively correlated with embryo quality. This was evidenced by reduced good-quality embryo rates and poorer clinical pregnancy and live birth outcomes among these women. These findings suggest that high leptin levels impair granulosa cell function, leading to compromised embryo development and fertility outcomes​.

### Dietary Interventions and Ovarian Morphology

Kazemi et al. [17] reported that higher adherence to dietary quality indices, such as the Mediterranean and Dietary Approaches to Stop Hypertension (DASH) diets, was associated with improved ovarian morphology, as reflected in decreased ovarian volume (OV) and follicle number per ovary (FNPO). These effects were mediated through reductions in obesity BMI and waist circumference (WC), insulin resistance, and hyperandrogenism. The study emphasizes the potential of dietary interventions in mitigating obesity-related ovarian dysfunction​.

### Ovarian Antral Follicle Dynamics and Endocrine Function

Oldfield et al. [18] found that women with obesity exhibited significant alterations in ovarian antral follicle dynamics, including reduced recruitment events, fewer selectable follicles, and lower anti-Müllerian hormone (AMH) levels. Additionally, luteal phase defects were markedly more prevalent among women with obesity, affecting 76% compared to 29% in non-obese women. These disruptions occurred despite regular menstrual cycles, indicating that obesity suppresses ovarian function at multiple levels​.

#### Impact of Obesity on Male Fertility

Obesity detrimentally affects male fertility through alterations in hormonal balance, sperm quality, and molecular characteristics, as well as by influencing early embryonic development.

### Hormonal Dysregulation

Bieniek et al. [19] found that men with obesity exhibited significant reductions in total testosterone, free testosterone, and sex hormone-binding globulin (SHBG) levels, alongside elevated estradiol levels. However, semen parameters such as volume, count, motility, and morphology remained unaffected across BMI categories. These findings suggest a strong link between obesity-induced hypogonadism and male reproductive dysfunction​.

### Decline in Sperm Quality

Obesity was associated with poorer sperm motility and increased DNA fragmentation. Kort et al. [20] observed that men with obesity had significantly fewer normal-motile sperm and higher sperm DNA fragmentation indices (DFI). Similarly, La Vignera et al. [21] reported increased sperm apoptosis, chromatin decondensation, and DNA fragmentation among men with obesity, emphasizing the adverse impact of elevated BMI on both conventional and non-conventional sperm parameters​​.

### Semen Function and Epididymal Activity

Martini et al. [22] highlighted that obesity negatively affects total and rapid sperm motility, as well as epididymal function, measured by N-acetyl-glucosaminidase (NAG) levels. Seminal fructose levels were positively associated with BMI, but sperm morphology, concentration, and testosterone levels remained unchanged.

### Age-BMI Interaction in Sperm Production

Paasch et al. [23] reported that higher BMI was correlated with reduced total sperm count (TSC), total cell number (TCN), testosterone levels, and inhibin-B concentrations. The effects of BMI were most pronounced in younger men aged 20–30, where obesity significantly impacted TSC and TCN.

### Molecular Effects and Embryo Development

Raad et al. [24] revealed that obesity adversely affects sperm chromatin integrity, DNA methylation, and oxidative stress levels. These molecular abnormalities were linked to delayed embryonic cell cycles (CC1, CC3), indicating that paternal obesity compromises early embryonic development.

#### Impact of Obesity on ART Outcomes

Obesity is a well-documented risk factor negatively influencing outcomes in ART. Its effects span reduced ovarian response, compromised oocyte and embryo quality, impaired uterine receptivity, and altered metabolic conditions, ultimately leading to lower pregnancy and live birth rates.

### Ovarian Response and Stimulation

Women with obesity undergoing ART require higher gonadotropin doses and longer stimulation durations. Pinborg et al. [25] and Devranoğlu et al. [26] reported fewer retrieved oocytes and lower embryo yields in women with obesity. These patients also demonstrated lower live birth rates​​. Caillon et al. [27] emphasized that while obesity does not inherently compromise ovarian stimulation when protocols are adjusted, it results in increased transfer cancellations and miscarriage rates​. Haghighi et al. [28] suggested that weight loss prior to ART could mitigate obstetric complications without significantly altering ovarian response​.

### Oocyte and Embryo Quality

Obesity impacts on oocyte morphology and embryo development. Leary et al. [29] highlighted smaller oocytes altered glucose metabolism, and higher triglyceride levels in embryos from women with obesity, impairing blastocyst quality​. Bellver et al. [30] observed a reduction in implantation and live birth rates in ovum donation recipients with obesity, implicating poor uterine receptivity and systemic metabolic dysfunction. Zhang et al. [31] showed minimal stimulation IVF may preserve oocyte quality better than conventional protocols in patients with obesity​.

### Uterine Receptivity and Implantation

Obesity adversely affects uterine receptivity. Luke et al. [32] found higher rates of implantation failure and reduced live birth rates in women with obesity using autologous oocytes, with donor cycles showing no such disparities, confirming uterine involvement​. Bellver et al. [30] further underscored the role of obesity in reducing implantation rates across BMI categories​.

### Pregnancy and Live Birth Outcomes

Obesity is associated with lower live birth rates and higher miscarriage risks. Veleva et al. [33] and Chavarro et al. [34] reported significantly reduced live birth rates in women with obesity undergoing ART, alongside increased miscarriage risks. These adverse outcomes persisted despite weight loss interventions​​. Kim et al. [35] linked obesity to diminished ovarian reserve, but not to gamete production in ART​.

### Metabolic Syndrome and ART Challenges

MetS exacerbates poor ART outcomes. He et al. [36] noted poorer ovarian response, fewer retrieved embryos, and reduced cumulative live birth rates in women with MetS undergoing ART, necessitating metabolic management prior to treatment​. Kudesia et al. [37] showed increased cycle cancellations and fewer usable embryos in women with obesity, particularly those with PCOS.

### Tailored ART Protocols

Personalized approaches may alleviate some adverse effects. Orvieto et al. [38] demonstrated that patients with obesity achieved comparable oocyte and embryo quality to non-obese patients when large follicles (> 15 mm) were aspirated, suggesting that stimulation response, rather than intrinsic oocyte quality, is the limiting factor​. Legge et al. [39] reported no significant differences in clinical pregnancy or live birth rates between BMI groups when protocols were adjusted, highlighting the importance of individualized stimulation​.

#### Maternal and Neonatal Outcomes Associated with Obesity During Pregnancy

Obesity during pregnancy is a significant risk factor for adverse maternal and neonatal outcomes. These outcomes include gestational complications, altered delivery modes, and neonatal metabolic and physical health risks.

### Maternal Complications

Obesity increases the likelihood of gestational hypertension, diabetes, and excessive gestational weight gain. Aly et al. [40] and Joy et al. [41] reported that maternal obesity significantly raises the risks of hypertension, gestational diabetes mellitus (GDM), and cesarean delivery, with severity of obesity correlating with increased risks​​. Sabolović Rudman et al. [42] observed that women with obesity and GDM experienced higher gestational weight gain and hypertension rates compared to their normal-weight counterparts​. Shen et al. [43] highlighted prepregnancy obesity as a key predictor of gestational weight gain and cesarean delivery, emphasizing the need for early intervention​.

### Mode of Delivery

Obesity is a leading factor for increased cesarean delivery rates: Magriples et al. [44] noted that women with obesity and overweight women had higher cesarean delivery risks, independent of gestational weight gain or infant size​. Melchor et al. [45] confirmed a strong association between obesity and cesarean delivery, with an adjusted odds ratio (aOR) of 2.76 for women with obesity compared to those with normal BMI​.

### Neonatal Outcomes

Obesity influences neonatal health through macrosomia, increased neonatal intensive care unit (NICU) admissions, and altered metabolic profiles. Dickey et al. [46] reported that maternal BMI affects neonatal outcomes differently in singleton versus twin pregnancies, with singletons showing a higher risk of macrosomia while NICU admission rates were elevated across all BMI groups in twins​. Adwani et al. [47] noted higher rates of macrosomia and perineal injuries in women with obesity, particularly in class III obesity​. Melchor et al. [45] observed a two- to threefold increase in macrosomia rates among mothers with obesity, with associated neonatal complications such as higher NICU admissions​.

### Risk of Miscarriage

Obesity is linked to increased miscarriage risk, particularly in the first trimester. Bhandari et al. [48] found a higher likelihood of early pregnancy losses in women with obesity with recurrent miscarriage, often linked to metabolic and endometrial dysfunction​.

### Impact of Gestational Diabetes Mellitus

Obesity exacerbates the adverse effects of GDM. Yogev et al. [49] observed that while obesity and morbid obesity both impair pregnancy outcomes, insulin treatment improves results compared to diet-only interventions​.

### Preterm Birth

The relationship between obesity and preterm birth is complex. Dickey et al. [46] reported that obesity in singleton pregnancies reduces preterm birth risks, but the effect does not extend to twin pregnancies​. Shen et al. [43] and Melchor et al. [45] found no significant difference in preterm birth rates across BMI groups.

**Fig. 2:**
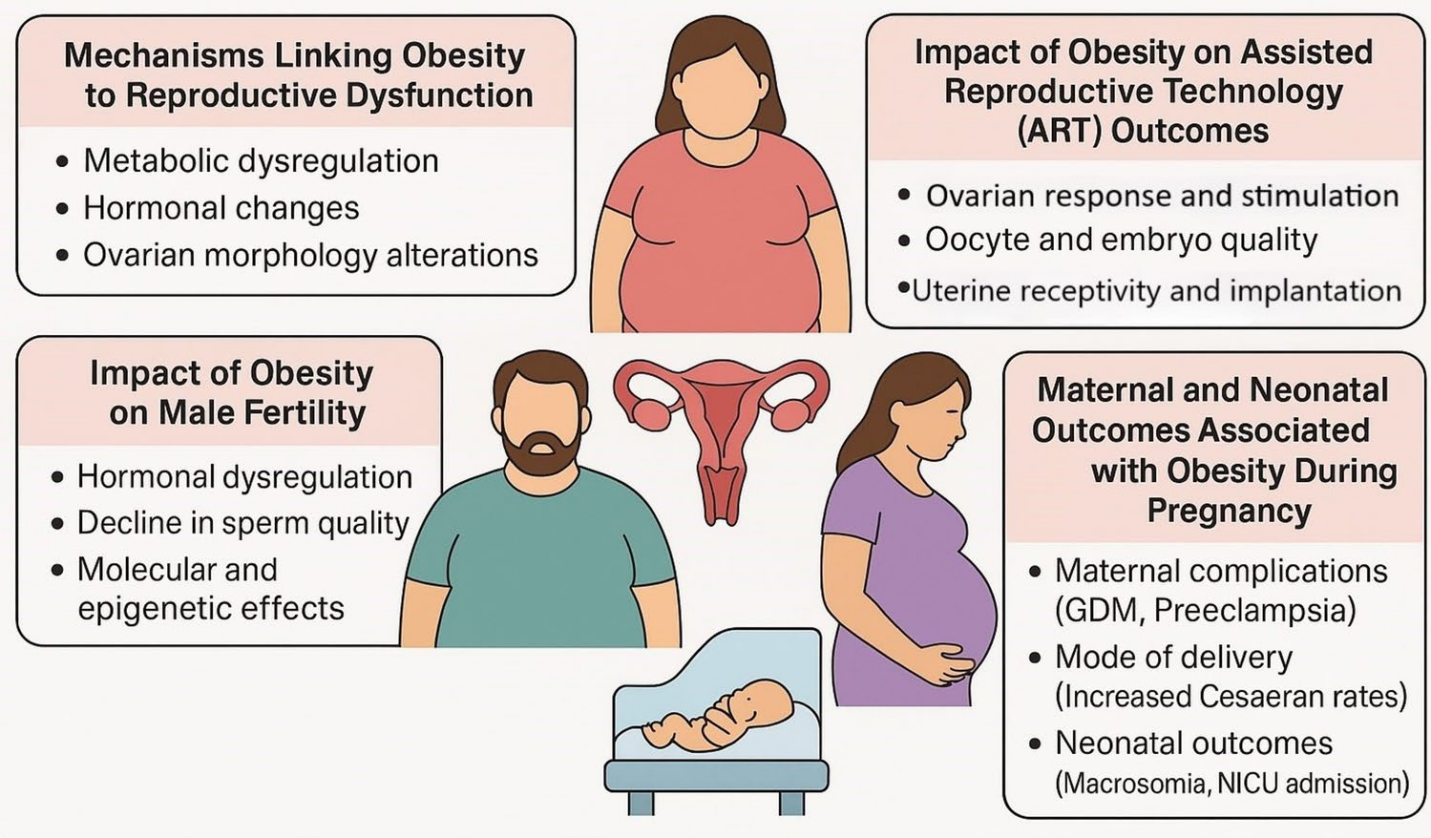
Multifaceted Effects of Obesity on Male and Female Reproductive Function, ART Outcomes, and Pregnancy-Related Complications. ART: assisted reproductive technique; GDM: gestational diabetes mellitus; NICU: neonatal intensive care unit

Figure 2 summarizes the multifaceted effects of obesity on male and female reproductive function, ART outcomes, and pregnancy-related complications.

## Discussion

This systematic review highlights the significant impact of obesity on reproductive health, ART outcomes, and pregnancy-related maternal and neonatal risks. The findings reveal that obesity disrupts reproductive health through multifactorial mechanisms, including metabolic dysregulation, hormonal imbalances, and alterations in ovarian function. These disruptions manifest as impaired ovulatory function, reduced oocyte quality, and compromised embryo development, collectively contributing to diminished fertility. Notably, MetS, frequently associated with obesity, exacerbates these effects by prolonging TTP and increasing infertility risk, independent of overall obesity levels. Elevated triglycerides and reduced HDL cholesterol, key metabolic markers of MetS, further underscore the role of specific metabolic abnormalities in delayed fertility and suboptimal reproductive outcomes.

The influence of obesity extends significantly to ART outcomes, where it negatively affects every stage of the process. Women with obesity demonstrate poorer ovarian response to stimulation, requiring higher gonadotropin doses and longer treatment durations. This often results in lower oocyte retrieval rates and reduced embryo yields. Furthermore, the quality of oocytes and embryos is compromised, with altered glucose metabolism and developmental abnormalities contributing to lower implantation and live birth rates. The detrimental effects of obesity also extend to uterine receptivity, where implantation failure and increased miscarriage risks are observed, even among recipients of donor oocytes, highlighting the systemic impact of obesity on reproductive potential.

Obesity also poses substantial risks during pregnancy, both for the mother and the neonate. Maternal complications, including GDM, preeclampsia, and increased cesarean delivery rates, are markedly higher in individuals with obesity. Excessive gestational weight gain and associated hypertension further exacerbate these risks, necessitating careful monitoring and management. Neonates born to mothers with obesity are at heightened risk for adverse outcomes, including macrosomia, increased rates of NICU admission, and long-term metabolic disturbances. These findings underscore the intergenerational effects of obesity, perpetuating cycles of health challenges that affect both maternal and offspring well-being.

In clinical practice, male obesity-related subfertility warrants greater attention. Hormonal imbalances, such as reduced testosterone and elevated estradiol levels, along with increased sperm DNA fragmentation and impaired chromatin integrity, suggest that male partners should undergo thorough reproductive evaluations when obesity is present [19–24]. These findings highlight the importance of including men in preconception counseling and interventions, especially since weight loss and metabolic improvements may restore hormonal balance and improve sperm function. Addressing male factors is particularly critical in ART contexts, where paternal obesity can influence embryo quality and developmental kinetics, ultimately affecting implantation success and live birth outcomes.

Zheng et al. [7] highlighted the disruption of the hypothalamic-pituitary-ovarian (HPO) axis due to insulin resistance, chronic inflammation, and altered adipokine secretion, leading to ovulatory dysfunction and poor oocyte quality. This review emphasized the direct role of visceral adiposity in worsening metabolic dysfunction in conditions like PCOS​. Prodan et al. [11] expanded on these mechanisms, identifying the imbalance between pro- and anti-inflammatory cytokines, decreased SHBG levels, and heightened estrogen conversion as key mediators of reproductive dysfunction. The review stressed that these hormonal imbalances are compounded by obesity’s metabolic effects​.

Our review integrates these perspectives but delves further into the role of specific metabolic syndrome components (e.g., dyslipidemia, elevated triglycerides, and reduced HDL cholesterol) in prolonging TTP and increasing infertility risks. By linking these metabolic abnormalities to reproductive dysfunction, our review provides a more nuanced understanding of how obesity interacts with hormonal and metabolic pathways to impair fertility.

Barbagallo et al. [50] and Palmer et al. [10] focused on obesity’s effects on male ART outcomes, specifically sperm motility, DNA integrity, and epigenetic alterations. They argued that these factors impair embryo quality and decrease live birth rates, with obesity acting as a key modifiable factor in male infertility​​. Langley-Evans et al. [51] emphasized reduced ART success in women with obesity, linking poor ovarian response, lower implantation rates, and heightened miscarriage risks to hormonal and metabolic imbalances​. Prodan et al. [11] discussed technical challenges in ART among women with obesity, such as difficulty in retrieving high-quality oocytes due to poor ovarian stimulation and increased complications during embryo transfer​. Our review builds on these findings by integrating male and female perspectives. It identifies that women with obesity require higher gonadotropin doses, experience higher cycle cancellation rates, and exhibit reduced implantation and live birth rates. Additionally, our review emphasizes paternal contributions to ART outcomes, noting the role of sperm epigenetics in embryo development and offspring health. This comprehensive approach underscores the need for tailored ART protocols to address gender-specific and combined obesity-related challenges.

Catalano [52] identified preeclampsia, GDM, and cesarean delivery as major risks associated with maternal obesity. This study emphasized that these risks are exacerbated by excessive gestational weight gain and pre-existing metabolic conditions​. Langley-Evans et al. [51] supported these findings but expanded on the long-term consequences, such as increased postpartum weight retention and future pregnancy risks. The review highlighted that maternal obesity perpetuates a cycle of adverse outcomes through poor weight management during and after pregnancy​. Prodan et al. [11] added a clinical perspective, emphasizing the challenges of managing prenatal care in women with obesity, such as reduced ultrasound accuracy, higher cesarean rates, and complications like macrosomia and NICU admissions​. Our review corroborates these findings but contributes a critical perspective by emphasizing intergenerational impacts. We highlight how maternal obesity increases the risk of childhood obesity, metabolic syndrome, and cardiovascular disease in offspring. This broader lens integrates immediate maternal and neonatal outcomes with long-term health trajectories, illustrating the far-reaching implications of maternal obesity on public health.

Palmer et al. [10] and Barbagallo et al. [50] described how male obesity reduces testosterone levels, increases estrogen conversion, and impairs spermatogenesis. They noted that these effects are partly reversible through weight loss or bariatric interventions​​. Zheng et al. [7] highlighted the role of visceral fat in exacerbating male hypogonadism and sperm DNA damage, emphasizing the importance of adipokine imbalances and chronic inflammation in male infertility​. Our review aligns with these findings but goes a step further by exploring how sperm epigenetic modifications contribute to transgenerational health issues.

Craig et al. [53] provided a focused analysis of obesity’s impact on male fertility, emphasizing hormonal disruptions, such as hypogonadotropic hypogonadism and hyperestrogenism, alongside epigenetic alterations in sperm that affect both fertility and offspring health. This review highlighted the role of obesity-related comorbidities like diabetes and sleep apnea in exacerbating male infertility and discusses interventions, including weight loss and bariatric surgery, while noting potential risks of rapid weight reduction. In contrast, our review offers a broader scope, integrating male and female reproductive challenges, ART outcomes, and pregnancy complications. While Craig et al. [53] centered on male contributions to ART success, our review goes further by addressing the compounded effects of obesity in couples, linking metabolic syndrome to infertility, and proposing tailored ART protocols. Additionally, our review expands the discussion on intergenerational impacts by synthesizing both paternal and maternal contributions, providing a more comprehensive and practical framework for managing obesity in reproductive health.

Catalano [52] and Langley-Evans et al. [51] emphasized preconception lifestyle interventions, such as weight loss, dietary modification, and exercise, as effective strategies for improving reproductive and pregnancy outcomes. Both reviews advocate for structured antenatal care to mitigate risks during pregnancy​​. Prodan et al. [11] underscored the importance of individualized antenatal care, particularly for managing obesity-related pregnancy risks. The review suggested that integrating nutritional counseling and closer monitoring into routine care could improve maternal and neonatal outcomes​. Our review supports these recommendations but also highlights the need for tailored ART protocols to address obesity-specific challenges in both men and women. By combining lifestyle interventions with personalized clinical strategies, our review presents a dual approach that bridges prevention and treatment, offering practical solutions for managing obesity in reproductive health.

Our review is comprehensive in scope, addressing obesity's impact on male and female reproductive health, ART outcomes, and pregnancy-related risks, providing a holistic perspective. It incorporates data from diverse populations and regions, enhancing the global relevance of findings. By integrating both male and female perspectives, it highlights the broader implications of obesity on fertility and reproductive success. The detailed exploration of physiological mechanisms, including hormonal, metabolic, and epigenetic pathways, is complemented by clinically relevant insights for preconception care, ART protocols, and antenatal management. The rigorous methodology, adhering to PRISMA guidelines and robust quality assessment tools, ensures the reliability of the findings. Furthermore, the review identifies critical gaps in the literature and proposes future research directions, making it a valuable resource for advancing clinical practice and guiding future studies.

Our review has some limitations. The studies included in our systematic review varied significantly in their design, population characteristics, and definitions of obesity. This heterogeneity may affect the comparability of results and limit the strength of synthesized conclusions. Despite efforts to include diverse studies, there was limited representation of men and populations from low- and middle-income countries. This underrepresentation restricts the generalizability of findings to these groups, which may face unique challenges related to obesity and reproductive health. Several included studies had small sample sizes or methodological heterogeneity in measuring outcomes such as ART success rates or neonatal health. This may reduce the statistical power and reliability of the findings synthesized in our review. While observational studies provide valuable insights, their design limits the ability to infer causation. RCTs were less represented, which might have provided stronger evidence for some findings. Our review may be subject to publication bias, as studies with statistically significant results are more likely to be published. This could lead to an overrepresentation of positive findings. Another key limitation was the heterogeneity in definitions and measurement criteria across studies. For instance, BMI cut-off points for classifying obesity varied slightly by region or study protocol, and diagnostic criteria for conditions like metabolic syndrome or PCOS were not uniform. Similarly, outcome measures for ART success and fertility metrics were not always consistently reported. These methodological inconsistencies may limit comparability across studies and introduce variability in the strength and direction of observed associations.

### Clinical Implications

The findings underscore the critical need for preconception weight management strategies to improve reproductive health outcomes. Structured lifestyle interventions, including dietary changes, physical activity, and behavioral counseling, should be integral to preconception care programs. For individuals with severe obesity or metabolic syndrome, bariatric surgery or pharmacological interventions may be considered as part of a comprehensive approach to enhance fertility and optimize ART outcomes. Screening and management of metabolic dysfunction, including insulin resistance, dyslipidemia, and hormonal imbalances, are essential to mitigate the adverse effects of obesity on reproductive health. Clinicians should focus on personalized treatment plans addressing these factors, particularly in patients with PCOS or metabolic syndrome.

Early screening and intervention for GDM and hypertensive disorders are critical in obese pregnancies. Tailored nutritional counseling, glucose monitoring, and, when necessary, pharmacological management (e.g., insulin therapy for GDM) should be implemented to minimize maternal and neonatal complications. Obese pregnancies require enhanced prenatal care, including frequent monitoring of fetal growth and maternal health, specialized ultrasound techniques to address technical challenges, and individualized delivery planning to reduce the risk of cesarean delivery and related complications.

### Future Research Directions

Further investigation is needed to elucidate the molecular pathways linking obesity to male infertility. This includes understanding the role of epigenetic modifications, oxidative stress, and hormonal disruptions in sperm dysfunction and embryonic development. Research should focus on developing tailored ART protocols that address the unique challenges posed by obesity. This includes optimizing gonadotropin dosing, improving embryo culture conditions, and integrating metabolic interventions to enhance ART success rates in both men and women. Long-term, multicentric studies are necessary to examine the intergenerational consequences of obesity on reproductive and metabolic health. These studies should assess how maternal and paternal obesity impact offspring health outcomes, including fertility, metabolic syndrome, and chronic disease risk.

## Conclusions

This systematic review highlights the multifaceted impact of obesity on reproductive health, ART outcomes, and pregnancy-related risks. Obesity disrupts reproductive function through hormonal, metabolic, and epigenetic pathways, leading to reduced fertility, suboptimal ART outcomes, and increased risks of maternal and neonatal complications. The findings emphasize the critical need for preconception interventions, including weight management and metabolic optimization, to mitigate these adverse effects. Tailored ART protocols and enhanced antenatal care for patients with obesity are essential to improving reproductive and pregnancy outcomes. Clinicians should also incorporate targeted screening and counseling for men with obesity as part of preconception care, given its measurable impact on sperm quality and embryonic development. Additionally, the review underscores the importance of addressing intergenerational health implications, as maternal and paternal obesity contribute to long-term metabolic and reproductive challenges in offspring. By identifying gaps in the current literature and proposing future research directions, this review provides a foundation for advancing clinical strategies and guiding future investigations in the field.

## Key References

- Oldfield AL, Vanden Brink H, Carter FE, Jarrett BY, Lujan ME. Obesity is associated with alterations in antral follicle dynamics in eumenorrheic women. Hum Reprod. 2023 Mar 1;38(3):459–470.10.1093/humrep/dead007.
  - This study offers critical insights into how obesity alters ovarian antral follicle dynamics, including recruitment, dominance, and selection, even in women with regular menstrual cycles. It highlights the intricate effects of obesity on ovarian function, making it a valuable contribution to understanding subfertility mechanisms.
- Devranoğlu B, Yilmaz MB, Peker G, Emekçi Özay Ö, Özay AC, Güzel Aİ. Effects of obesity on clinical outcomes in diminished ovarian reserve patients undergoing intracytoplasmic sperm injection cycles. Medicine (Baltimore). 2024;103(28):e38942. 10.1097/MD.0000000000038942.
  - This study investigates the effects of obesity on ovarian response in patients with diminished ovarian reserve (DOR) undergoing intracytoplasmic sperm injection (ICSI). It underscores the importance of personalized stimulation protocols to optimize reproductive outcomes in this vulnerable population.
- Schon SB, Cabre HE, Redman LM. The impact of obesity on reproductive health and metabolism in reproductive-age females. Fertil Steril 2024;122(2):194–203. 10.1016/j.fertnstert.2024.04.036.
  - This recent review comprehensively examines the impact of obesity on reproductive health and metabolism in reproductive-age females. It offers new perspectives on hormonal imbalances and their implications for fertility, enhancing the understanding of obesity-related reproductive challenges.
- Zheng L, Yang L, Guo Z, Yao N, Zhang S, Pu P. Obesity and its impact on female reproductive health: unraveling the connections. Front Endocrinol (Lausanne). 2024 Jan 9;14:1326546. 10.3389/fendo.2023.1326546.
  - This study provides robust evidence on how obesity disrupts the hypothalamic-pituitary-ovarian (HPO) axis through insulin resistance, chronic inflammation, and adipokine secretion. The detailed analysis of metabolic dysfunction mechanisms significantly advances the field of reproductive endocrinology.
- He Y, Lu Y, Zhu Q, Wang Y, Lindheim SR, Qi J, Li X, Ding Y, Shi Y, Wei D, Chen ZJ, Sun Y. Influence of metabolic syndrome on female fertility and in vitro fertilization outcomes in PCOS women. Am J Obstet Gynecol. 2019;221(2):138.e1 - 138.e12. 10.1016/j.ajog.2019.03.011.
  - This secondary analysis of an RCT highlights how metabolic syndrome (MetS) exacerbates poor ART outcomes in women with PCOS. It calls for preconception metabolic management to enhance fertility and live birth rates, emphasizing the need for integrated care approaches.

## Supplementary Information

Below is the link to the electronic supplementary material.

Supplementary file1 (DOCX 61 KB)

## Data Availability

No datasets were generated or analysed during the current study.
