# Supplementary material for: When Weight Matters: How Obesity Impacts Reproductive Health and Pregnancy-A Systematic Review
Source: Curr Obes Rep. 2025 Apr 16;14(1):37. doi: 10.1007/s13679-025-00629-9 (PMC12003489; doi:10.1007/s13679-025-00629-9)
Supplement: Supplementary file 1 — Supplementary file1 (DOCX 61 KB) [file 13679_2025_629_MOESM1_ESM.docx]

**QUALITY ASSESSMENT**

**Study 1: Adwani et al:**

1. Selection of Study Groups

- Representativeness of the exposed cohort:
  The study included obese pregnant women attending a single tertiary hospital (King Khaled Hospital). While this ensures a consistent clinical setting, it may not represent all obese pregnant women across Saudi Arabia.
  Score: 1 (Somewhat representative).
- Selection of the non-exposed cohort:
  The study lacked a normal-weight control group, which is critical for comparative outcomes.
  Score: 0 (No control group).
- Ascertainment of exposure:
  Exposure (obesity) was clearly defined using BMI categories and confirmed via medical records.
  Score: 1 (Secure records).
- Demonstration that outcome of interest was not present at the start of the study:
  The study excluded women with pre-existing diabetes, hypertension, and other conditions to avoid confounding.
  Score: 1.

2. Comparability of Groups

- Comparability based on design or analysis:
  No adjustments were made for key confounding factors (e.g., socioeconomic status, gestational weight gain), which could impact pregnancy and neonatal outcomes.
  Score: 0.

3. Outcome Assessment

- Assessment of outcomes:
  Outcomes (maternal and neonatal complications) were assessed retrospectively using medical records, which are reliable but may lack detailed contextual data.
  Score: 1.
- Follow-up duration:
  The study followed participants until delivery, but no data were available on postpartum outcomes or long-term neonatal impacts.
  Score: 0 (Follow-up duration inadequate for key outcomes).
- Adequacy of follow-up of cohorts:
  The study excluded many participants due to missing data, reducing the overall sample size (186 participants). However, it is unclear if missing data biased the findings.
  Score: 0 (High loss due to missing data).

Total NOS Score: 4/9

- Interpretation:
  The study demonstrates moderate risk of bias due to:
  - Lack of a non-exposed control group.
  - Limited adjustment for confounders.
  - High loss of data and short follow-up duration

**Study 2: Bellver et al**

1. Selection of Study Groups

- Representativeness of the exposed cohort:
  The study includes recipients of ovum donations stratified by BMI into lean, normal, overweight, and obese groups. All donors were normoweight, ensuring uniformity in donor selection. The inclusion of over 9,000 cycles provides strong representativeness for recipients undergoing ovum donation.
  Score: 1 (Truly representative).
- Selection of the non-exposed cohort:
  Normoweight recipients serve as the reference group, allowing direct comparison across BMI categories.
  Score: 1.
- Ascertainment of exposure:
  BMI of recipients was calculated using standard methods, and exclusions were made for known uterine pathologies or other confounding conditions.
  Score: 1 (Secure records).
- Demonstration that outcome of interest was not present at the start of the study:
  The exclusion of recipients with conditions such as uterine pathology and recurrent miscarriages ensures outcomes were not present at baseline.
  Score: 1.

2. Comparability of Groups

- Comparability based on design or analysis:
  The study adjusts for some confounders (e.g., recipient age) in regression analyses, but does not fully account for factors like smoking status or PCOS, which are noted as limitations.
  Score: 1 (Adjusted for key confounders).

3. Outcome Assessment

- Assessment of outcomes:
  Outcomes, including implantation, clinical pregnancy, and live birth rates, were assessed using standardized clinical methods (e.g., b-HCG levels, ultrasound).
  Score: 1.
- Follow-up duration:
  The study followed pregnancies until live birth, providing complete data for key outcomes.
  Score: 1.
- Adequacy of follow-up of cohorts:
  The large sample size (9,587 cycles) and systematic data collection ensure minimal bias due to loss to follow-up.
  Score: 1.

Total NOS Score: 8/9

- Interpretation:
  This study demonstrates a low risk of bias, with robust methodology and representativeness. However, the absence of full adjustment for all potential confounders slightly lowers its score.

**Study 3: Bieniek et al.**

1. Selection of Study Groups

- Representativeness of the exposed cohort:
  The study evaluated 4,440 men from infertility clinics across multiple institutions in North America. While comprehensive, the cohort is inherently biased toward subfertile men and may not represent the general male population.
  Score: 1 (Somewhat representative).
- Selection of the non-exposed cohort:
  There was no explicit non-exposed comparison group (e.g., fertile men without obesity). Instead, comparisons were made between BMI categories (normal weight, overweight, and obese).
  Score: 0 (No distinct non-exposed group).
- Ascertainment of exposure:
  BMI was calculated using height and weight, with some measurements self-reported. While this is standard practice, self-reported data could introduce inaccuracies.
  Score: 1.
- Demonstration that outcome of interest was not present at the start of the study:
  The study included men undergoing fertility evaluation, and baseline reproductive parameters were measured. However, no data confirm whether reproductive outcomes were entirely unaffected at the start.
  Score: 0.

2. Comparability of Groups

- Comparability based on design or analysis:
  The study adjusted for confounders such as age and study center in multivariate models but did not account for other potential confounders, such as comorbidities or lifestyle factors.
  Score: 1.

3. Outcome Assessment

- Assessment of outcomes:
  Outcomes such as reproductive hormone levels and semen parameters were measured using standardized methods, ensuring reliability.
  Score: 1.
- Follow-up duration:
  The study assessed only cross-sectional data at the time of the fertility evaluation, lacking follow-up on outcomes such as pregnancy or live births.
  Score: 0 (No follow-up).
- Adequacy of follow-up of cohorts:
  As this was a cross-sectional study, follow-up adequacy is not applicable. However, data completeness for most participants ensures reliable conclusions.
  Score: 1.

Total NOS Score: 5/9

- Interpretation:
  The study has a moderate risk of bias due to the absence of a non-exposed cohort, reliance on cross-sectional data, and limited adjustment for potential confounders. Despite these limitations, it provides valuable insights into the relationship between BMI and male fertility parameters.

**Study 4: Chavarro et al.**

Selection of Study Groups

- Representativeness of the exposed cohort:
  The study included women undergoing ART cycles, primarily from a single fertility center in Massachusetts. While the cohort is highly specific to women seeking ART, it may not represent all women undergoing fertility treatments across other centers or demographics.
  Score: 1 (Somewhat representative).
- Selection of the non-exposed cohort:
  The study stratified participants based on BMI and weight change but lacked a distinct non-exposed or external reference group.
  Score: 0.
- Ascertainment of exposure:
  BMI and weight changes were objectively measured by trained research nurses, ensuring reliability in exposure assessment.
  Score: 1 (Secure records).
- Demonstration that outcome of interest was not present at the start of the study:
  Outcomes such as live birth and clinical pregnancy were assessed after ART cycles, ensuring these outcomes were absent at baseline.
  Score: 1.

2. Comparability of Groups

- Comparability based on design or analysis:
  The study adjusted for potential confounders, such as age, Day 3 FSH levels, infertility diagnosis, and treatment protocols. However, the impact of lifestyle factors and comorbidities (e.g., PCOS) may not have been fully accounted for.
  Score: 1.

3. Outcome Assessment

- Assessment of outcomes:
  ART outcomes, such as oocyte retrieval, fertilization rate, clinical pregnancy, and live birth, were assessed using standard clinical protocols, ensuring reliability.
  Score: 1.
- Follow-up duration:
  Follow-up was limited to the ART cycle, with no long-term outcomes assessed, such as neonatal health or postpartum changes.
  Score: 0 (Short follow-up duration).
- Adequacy of follow-up of cohorts:
  Most participants completed their ART cycles, and outcome data were available for 233 cycles. Loss to follow-up or missing data does not appear to bias findings significantly.
  Score: 1.

Total NOS Score: 6/9

- Interpretation:
  The study demonstrates moderate risk of bias, with strong methodologies in exposure and outcome assessments but limitations due to the absence of a distinct non-exposed cohort and short-term follow-up.

**Study 5: Devranoğlu et al.**

1. Selection of Study Groups

- Representativeness of the exposed cohort:
  The study included patients with diminished ovarian reserve (DOR) undergoing intracytoplasmic sperm injection (ICSI) cycles at a tertiary hospital in Turkey. While specific to this population, the study may not fully represent all patients with DOR due to geographical and institutional variability.
  Score: 1 (Somewhat representative).
- Selection of the non-exposed cohort:
  The study stratified patients into obese (BMI ≥ 30) and non-obese (BMI < 30) groups, allowing for direct comparison.
  Score: 1.
- Ascertainment of exposure:
  BMI was measured using standardized methods, ensuring reliable exposure assessment.
  Score: 1 (Secure records).
- Demonstration that outcome of interest was not present at the start of the study:
  Outcomes such as clinical pregnancy and oocyte maturity were assessed after ICSI cycles, ensuring these outcomes were not present at baseline.
  Score: 1.

2. Comparability of Groups

- Comparability based on design or analysis:
  The study accounted for confounders such as age, baseline ovarian reserve parameters (e.g., antral follicle count, FSH), and stimulation protocol. However, other potential confounders like lifestyle factors were not considered.
  Score: 1.

3. Outcome Assessment

- Assessment of outcomes:
  Outcomes, including clinical pregnancy and number of mature oocytes, were assessed using standardized protocols and objective measurements.
  Score: 1.
- Follow-up duration:
  The study followed patients only during the ICSI cycles, with no long-term outcomes such as live birth or neonatal health assessed.
  Score: 0 (Short follow-up duration).
- Adequacy of follow-up of cohorts:
  Data completeness appears high, with no indication of significant loss to follow-up.
  Score: 1.

Total NOS Score: 7/9

- Interpretation:
  The study demonstrates a low to moderate risk of bias, with strengths in its cohort selection and outcome assessment. However, it is limited by its short-term follow-up and lack of adjustment for certain confounders.

**Study 6: Dickey et al.**

**1. Selection of Study Groups**

- **Representativeness of the exposed cohort:**
  The study used a large dataset from the Society for Assisted Reproductive Technology Clinic Outcome Reporting System (SART CORS) with 56,556 singleton and 23,804 twin live births from fresh nondonor IVF cycles. This cohort is representative of patients undergoing assisted reproductive technology (ART) in the U.S.
  **Score: 1 (Truly representative).**
- **Selection of the non-exposed cohort:**
  No explicit non-exposed cohort was included, as the study focused on comparisons within exposed groups based on height, weight, and BMI.
  **Score: 0.**
- **Ascertainment of exposure:**
  Maternal height and weight were measured or reported prior to ART. The data were reliable, but missing values led to exclusions.
  **Score: 1.**
- **Demonstration that outcome of interest was not present at the start of the study:**
  Gestational age and outcomes (e.g., preterm birth) were assessed after ART procedures, ensuring these outcomes were absent at baseline.
  **Score: 1.**

**2. Comparability of Groups**

- **Comparability based on design or analysis:**
  The study adjusted for potential confounders, including maternal age, race, gravidity, smoking, use of ICSI, and number of embryos transferred. This ensures better comparability between BMI and height subgroups.
  **Score: 1 (Adjusted for key confounders).**

**3. Outcome Assessment**

- **Assessment of outcomes:**
  Preterm birth outcomes were rigorously defined, with gestational age verified against birth weight and other parameters. However, self-reported maternal height and weight might introduce some bias.
  **Score: 1.**
- **Follow-up duration:**
  The study did not extend follow-up beyond birth outcomes, limiting insights into long-term neonatal or maternal health effects.
  **Score: 0 (Short follow-up duration).**
- **Adequacy of follow-up of cohorts:**
  The dataset excluded cases with missing or outlier height and weight values, but gestational age distributions were similar between included and excluded records, minimizing selection bias.
  **Score: 1.**

**Total NOS Score: 7/9**

- **Interpretation:**
  This study demonstrates a low risk of bias, with strengths in cohort representativeness, exposure measurement, and statistical adjustments. The main limitations are the absence of a distinct non-exposed group and short-term follow-up.

**Study 7. Dupont et al.**

Selection of Study Groups

- Representativeness of the exposed cohort:
  The study included 330 male partners of subfertile couples from four centers, focusing on the relationship between BMI and sperm DNA fragmentation. This cohort represents infertile male populations but may not fully generalize to the broader population of men with varying fertility statuses.
  Score: 1 (Somewhat representative).
- Selection of the non-exposed cohort:
  The study compared normal-weight, overweight, and obese men but did not include a completely non-exposed group (e.g., men without fertility issues).
  Score: 0.
- Ascertainment of exposure:
  BMI was calculated based on measured height and weight at the time of semen analysis, ensuring reliable exposure assessment.
  Score: 1 (Secure records).
- Demonstration that outcome of interest was not present at the start of the study:
  DNA fragmentation and sperm motility were measured during the study, ensuring these outcomes were absent at baseline.
  Score: 1.

2. Comparability of Groups

- Comparability based on design or analysis:
  The study adjusted for confounding variables such as age and smoking status, which are known to influence sperm DNA fragmentation.
  Score: 1 (Adjusted for key confounders).

3. Outcome Assessment

- Assessment of outcomes:
  Sperm DNA fragmentation was assessed using the TUNEL assay, a reliable method for measuring DNA damage. However, interlaboratory variability and differences in processing across the four centers could introduce bias.
  Score: 1.
- Follow-up duration:
  The study was cross-sectional and did not assess long-term reproductive outcomes, such as live births or miscarriage rates.
  Score: 0 (No long-term follow-up).
- Adequacy of follow-up of cohorts:
  Data completeness was not explicitly discussed, but the study seems to have included most eligible participants with minimal exclusions.
  Score: 1.

Total NOS Score: 6/9

- Interpretation:
  The study has a moderate risk of bias. While it effectively measures exposure and adjusts for confounders, its limitations include the absence of a non-exposed group and lack of long-term outcome data.

**Study 8: Kazemi et al.**

Selection of Study Groups

- Representativeness of the exposed cohort:
  The study included 111 reproductive-aged women from academic institutions in New York, recruited for four ongoing studies. The inclusion criteria were comprehensive, focusing on women with varying diet qualities and ovarian morphologies. However, the study may not fully generalize to all reproductive-aged women due to geographical and institutional selection.
  Score: 1 (Somewhat representative).
- Selection of the non-exposed cohort:
  The study lacked a clearly defined non-exposed comparison group (e.g., women not adhering to the Mediterranean or DASH diet patterns). Comparisons were made between dietary pattern scores and ovarian morphology within the same population.
  Score: 0.
- Ascertainment of exposure:
  Dietary quality was measured using validated tools such as HEI-2015, aHEI-2010, aMED, and DASH scores derived from a food frequency questionnaire (FFQ). These tools provide reliable measures of diet adherence.
  Score: 1 (Secure records).
- Demonstration that outcome of interest was not present at the start of the study:
  Outcomes such as ovarian volume and follicle number per ovary were assessed at the time of ultrasonography, ensuring these were not pre-existing conditions in the analysis.
  Score: 1.

2. Comparability of Groups

- Comparability based on design or analysis:
  The study adjusted for potential confounders such as age, race, and energy intake in the mediation analyses. However, factors like physical activity, smoking, and history of pregnancy or infertility, which could influence ovarian morphology, were not accounted for.
  Score: 1 (Adjusted for some confounders).

3. Outcome Assessment

- Assessment of outcomes:
  Outcomes were measured using standardized transvaginal ultrasonography protocols and validated markers (e.g., ovarian volume, follicle counts), ensuring reliable and reproducible data.
  Score: 1.
- Follow-up duration:
  The study was cross-sectional, limiting its ability to assess long-term reproductive or metabolic outcomes.
  Score: 0 (No follow-up).
- Adequacy of follow-up of cohorts:
  Data collection appears comprehensive, with exclusions clearly stated (e.g., poor ovarian visualization, implausible dietary data), minimizing bias due to incomplete data.
  Score: 1.

Total NOS Score: 6/9

- Interpretation:
  The study has a moderate risk of bias. Strengths include the use of validated tools for exposure and outcome assessment and adjustments for key confounders. Limitations include the lack of a non-exposed group and short-term analysis.

**Study 9: Kim et al.**

Selection of Study Groups

- Representativeness of the exposed cohort:
  The study prospectively included 2,013 couples undergoing infertility treatment at a large reproductive center. The inclusion of a broad range of BMI and body fat percentage (%BF) categories provides generalizability to infertile populations. However, it may not fully represent the broader population of couples attempting to conceive naturally.
  Score: 1 (Somewhat representative).
- Selection of the non-exposed cohort:
  The study lacked a clearly defined non-exposed control group (e.g., couples without infertility or with normal body composition). Comparisons were made within BMI and %BF categories.
  Score: 0.
- Ascertainment of exposure:
  Body composition was measured using bioelectric impedance analysis (BIA), a validated and precise method for assessing %BF. BMI was also measured and categorized per standard guidelines.
  Score: 1 (Secure records).
- Demonstration that outcome of interest was not present at the start of the study:
  Outcomes such as ovarian response and sperm parameters were assessed after body composition measurements, ensuring no outcomes were pre-existing.
  Score: 1.

2. Comparability of Groups

- Comparability based on design or analysis:
  The study adjusted for potential confounders, including age, PCOS diagnosis, and other clinical factors, ensuring fair comparisons between groups.
  Score: 1 (Adjusted for key confounders).

3. Outcome Assessment

- Assessment of outcomes:
  Outcomes such as ovarian reserve markers, response to gonadotropin stimulation, and sperm quality parameters (e.g., total motile sperm count) were measured using standardized clinical protocols, ensuring reliability.
  Score: 1.
- Follow-up duration:
  The study was limited to cross-sectional data collected during the IVF cycles, without long-term follow-up on pregnancy or live birth outcomes.
  Score: 0 (No long-term follow-up).
- Adequacy of follow-up of cohorts:
  Data completeness was high, with minimal exclusions or missing data noted.
  Score: 1.

Total NOS Score: 6/9

- Interpretation:
  The study demonstrates a moderate risk of bias. While it effectively measures exposure and adjusts for key confounders, it is limited by the absence of a non-exposed group and lack of follow-up on long-term reproductive outcomes.

**Study 10. Kort et al.**

Selection of Study Groups

- Representativeness of the exposed cohort:
  The study included 520 men undergoing routine semen analysis in a fertility center. These participants were classified into BMI categories (normal, overweight, and obese). However, the study population may not fully represent all men in the general population, as they were specifically seeking fertility evaluations.
  Score: 1 (Somewhat representative).
- Selection of the non-exposed cohort:
  The study compared groups with different BMI levels but lacked a true non-exposed group (e.g., individuals not undergoing fertility evaluation).
  Score: 0.
- Ascertainment of exposure:
  BMI was calculated using objectively measured height and weight, ensuring reliable exposure assessment.
  Score: 1 (Secure records).
- Demonstration that outcome of interest was not present at the start of the study:
  The outcomes (e.g., sperm quality, motility, chromatin integrity) were measured from semen samples collected after BMI classification, ensuring outcomes were not present prior to analysis.
  Score: 1.

2. Comparability of Groups

- Comparability based on design or analysis:
  The study did not adjust for confounding variables such as age, lifestyle factors, or comorbidities, which could influence sperm quality and chromatin integrity.
  Score: 0.

3. Outcome Assessment

- Assessment of outcomes:
  Outcomes were measured using validated and standardized methods (e.g., Tygerberg strict criteria for sperm morphology, SCSA for chromatin integrity), ensuring reliability.
  Score: 1.
- Follow-up duration:
  The study used cross-sectional data and did not follow participants for long-term reproductive or pregnancy outcomes.
  Score: 0 (No follow-up).
- Adequacy of follow-up of cohorts:
  The study reported comprehensive data for all 520 participants without evidence of missing or excluded data.
  Score: 1.

Total NOS Score: 5/9

- Interpretation:
  The study has a moderate risk of bias. While it effectively measures exposure and outcomes, limitations include the absence of a non-exposed group, lack of confounder adjustments, and no long-term follow-up.

**Study 11. Kudesia et al.**

Selection of Study Groups

- Representativeness of the exposed cohort:
  The study included 51,198 women who underwent their first autologous IVF cycle across 13 fertility centers in the USA. While the large sample size and multi-center nature enhance representativeness, the study is limited to women undergoing IVF, which may not represent the broader infertile population.
  Score: 1 (Somewhat representative).
- Selection of the non-exposed cohort:
  The study compared BMI categories (underweight, normal, overweight, and obese) but lacked a truly non-exposed comparison group, as all participants underwent IVF treatment.
  Score: 0.
- Ascertainment of exposure:
  BMI was calculated objectively using height and weight recorded before the IVF cycle, ensuring reliable assessment of exposure.
  Score: 1 (Secure records).
- Demonstration that outcome of interest was not present at the start of the study:
  Outcomes such as cycle cancellation, oocyte count, usable embryos, and clinical pregnancy were assessed after the IVF cycle began, ensuring these outcomes were not present at baseline.
  Score: 1.

2. Comparability of Groups

- Comparability based on design or analysis:
  The study adjusted for multiple potential confounders, including age, AMH, antral follicle count, gonadotropin dose, and infertility diagnosis. However, lifestyle factors such as smoking or physical activity were not controlled for, which may influence IVF outcomes.
  Score: 1 (Adjusted for key confounders).

3. Outcome Assessment

- Assessment of outcomes:
  Outcomes were assessed using standardized clinical definitions, such as clinical pregnancy confirmed via ultrasound and oocyte/embryo counts based on laboratory data. This ensures reliable measurements.
  Score: 1.
- Follow-up duration:
  The study only evaluated outcomes within the IVF cycle, without assessing live birth rates or longer-term maternal and neonatal health outcomes.
  Score: 0 (Short follow-up duration).
- Adequacy of follow-up of cohorts:
  There is no indication of significant loss to follow-up or missing data for the primary outcomes, ensuring reliable results.
  Score: 1.

Total NOS Score: 6/9

- Interpretation:
  The study demonstrates moderate risk of bias, with strengths in exposure and outcome assessment and confounder adjustment. Limitations include the lack of a non-exposed group and short-term follow-up.

**Study 12. La Vignera et al.**

1. Selection of Study Groups

- Representativeness of the exposed cohort:
  The study included 50 overweight, 50 obese, and 50 normal-weight healthy nonsmoking men randomly selected from the general population. This selection is representative of healthy men in this demographic but excludes other populations, such as smokers or individuals with underlying health conditions.
  Score: 1 (Somewhat representative).
- Selection of the non-exposed cohort:
  The study used normal-weight men as a control group, which provides a direct comparison to overweight and obese groups.
  Score: 1.
- Ascertainment of exposure:
  BMI was calculated using height and weight measurements, ensuring objective and reliable exposure assessment.
  Score: 1 (Secure records).
- Demonstration that outcome of interest was not present at the start of the study:
  All participants underwent a thorough diagnostic workup, including a medical history review, physical examination, and laboratory evaluations, ensuring no pre-existing andrological diseases known to affect sperm parameters.
  Score: 1.

2. Comparability of Groups

- Comparability based on design or analysis:
  The study controlled for potential confounding factors by including only nonsmoking, healthy men without systemic or andrological diseases. However, it did not account for lifestyle factors such as diet or physical activity, which may influence sperm parameters.
  Score: 1 (Adjusted for key confounders).

3. Outcome Assessment

- Assessment of outcomes:
  Conventional and non-conventional sperm parameters (e.g., mitochondrial membrane potential, chromatin compactness, DNA fragmentation) were measured using validated methods such as flow cytometry and WHO 1999 criteria. These are reliable and reproducible.
  Score: 1.
- Follow-up duration:
  The study was cross-sectional and did not follow participants for long-term reproductive outcomes, such as conception or live birth rates.
  Score: 0 (No follow-up).
- Adequacy of follow-up of cohorts:
  Data completeness was ensured for all 150 participants, with no indication of significant exclusions or missing data.
  Score: 1.

Total NOS Score: 7/9

- Interpretation:
  The study demonstrates a low risk of bias, with strengths in cohort selection, exposure and outcome measurement, and control for confounders. Its primary limitation is the lack of long-term follow-up on reproductive outcomes.

**Study 13. Leary et al.**

Selection of Study Groups

- Representativeness of the exposed cohort:
  The study included 29 overweight/obese and 29 normal-weight women undergoing fertility treatment, with embryos donated for research after clinical use. While this reflects women undergoing IVF/ICSI treatment, it may not represent the general population.
  Score: 1 (Somewhat representative).
- Selection of the non-exposed cohort:
  The study included a control group of normal-weight women (BMI 19–24.9), ensuring a comparison between exposed and non-exposed cohorts.
  Score: 1.
- Ascertainment of exposure:
  BMI was objectively measured during clinical treatment, ensuring reliable classification of exposure.
  Score: 1 (Secure records).
- Demonstration that outcome of interest was not present at the start of the study:
  Outcomes such as embryo metabolic and developmental parameters were assessed post-IVF, ensuring these were not present prior to treatment.
  Score: 1.

2. Comparability of Groups

- Comparability based on design or analysis:
  The study controlled for confounders such as male BMI, age, cause of infertility, and embryo grading in multivariate analyses. However, lifestyle factors were not accounted for, which could influence outcomes.
  Score: 1 (Adjusted for key confounders).

3. Outcome Assessment

- Assessment of outcomes:
  Outcomes such as metabolic profiles, embryo size, and cell counts were measured using validated techniques, including time-lapse imaging and biochemical assays, ensuring high reliability.
  Score: 1.
- Follow-up duration:
  The study did not follow embryos beyond the blastocyst stage, missing potential long-term reproductive or neonatal outcomes.
  Score: 0 (No follow-up).
- Adequacy of follow-up of cohorts:
  Data completeness was high, with embryos consistently followed up until developmental arrest or Day 9.
  Score: 1.

Total NOS Score: 7/9

- Interpretation:
  The study has a low risk of bias. Strengths include rigorous outcome measurement and confounder adjustment. However, limitations include lack of long-term follow-up and limited representativeness of the general population.

**Study 14: Legge et al.**

Selection of Study Groups

- Representativeness of the exposed cohort:
  The study included 752 women undergoing 951 IVF or IVF-ICSI cycles at a private fertility clinic in Halifax, Nova Scotia. The sample was stratified by BMI into normal weight, overweight, and obese categories. While the study population reflects women undergoing assisted reproduction, it may not fully generalize to all infertile women.
  Score: 1 (Somewhat representative).
- Selection of the non-exposed cohort:
  The study used normal-weight women (BMI < 25 kg/m²) as the reference group, providing a clear control group for comparison.
  Score: 1.
- Ascertainment of exposure:
  BMI was calculated based on measured weight and self-reported height, which is a reliable but not optimal method due to potential inaccuracies in self-reported height.
  Score: 1.
- Demonstration that outcome of interest was not present at the start of the study:
  Clinical outcomes such as cycle cancellation, pregnancy rates, and live birth were assessed after IVF treatment cycles, ensuring these outcomes were not pre-existing.
  Score: 1.

2. Comparability of Groups

- Comparability based on design or analysis:
  The study adjusted for confounders, including age, day 3 serum FSH level, duration of infertility, smoking status, and PCOS diagnosis. This ensures adequate control of major confounders.
  Score: 1 (Adjusted for key confounders).

3. Outcome Assessment

- Assessment of outcomes:
  IVF outcomes, including gonadotropin doses, cycle cancellation, clinical pregnancy, and live birth rates, were assessed using well-defined clinical criteria and standardized methods.
  Score: 1.
- Follow-up duration:
  The study was limited to assessing IVF outcomes within a single treatment cycle, with no follow-up on long-term reproductive or neonatal outcomes.
  Score: 0 (No long-term follow-up).
- Adequacy of follow-up of cohorts:
  Data completeness was high, with only 3.6% of cycles excluded due to missing BMI data. Sensitivity analyses confirmed the robustness of the findings.
  Score: 1.

Total NOS Score: 7/9

- Interpretation:
  This study demonstrates a low risk of bias, with strengths in exposure and outcome measurement and appropriate confounder adjustment. Its primary limitation is the lack of long-term follow-up for live birth outcomes and the reliance on self-reported height for BMI calculation.

**Study 15: Magriples et al.**

Selection of Study Groups

- Representativeness of the exposed cohort:
  The study included 841 young women (ages 14–25) receiving prenatal care in two urban clinics. Participants were stratified by BMI categories (underweight, normal weight, overweight, and obese). While the population represents urban, low-income settings, it may not be generalizable to other populations.
  Score: 1 (Somewhat representative).
- Selection of the non-exposed cohort:
  Normal-weight women (BMI 19.8–25.9) were used as the reference group, allowing comparisons between exposed and non-exposed groups.
  Score: 1.
- Ascertainment of exposure:
  Prepregnancy BMI was reported by participants and verified through medical records, ensuring a reliable measure of exposure.
  Score: 1 (Secure records).
- Demonstration that outcome of interest was not present at the start of the study:
  Outcomes such as cesarean delivery and infant birth weight were assessed postnatally, ensuring these outcomes were not pre-existing.
  Score: 1.

2. Comparability of Groups

- Comparability based on design or analysis:
  The study adjusted for potential confounders, including age, race, parity, smoking, alcohol use, and gestational age at delivery. This ensures robust comparisons across BMI categories.
  Score: 1 (Adjusted for key confounders).

3. Outcome Assessment

- Assessment of outcomes:
  Outcomes such as cesarean delivery and infant size (SGA, AGA, LGA) were assessed using standardized methods, including chart reviews and medical records. This ensures reliable outcome measurement.
  Score: 1.
- Follow-up duration:
  The study followed participants through delivery but did not assess long-term outcomes such as child health or maternal postpartum recovery.
  Score: 0 (No long-term follow-up).
- Adequacy of follow-up of cohorts:
  Data completeness was high, with over 95% of participants having complete records. There were no significant differences between those excluded and those included in the analysis.
  Score: 1.

Total NOS Score: 7/9

- Interpretation:
  This study demonstrates a low risk of bias, with strong methodologies for exposure and outcome assessment and appropriate confounder adjustment. The main limitation is the lack of long-term follow-up.

**Study 16: Melchor et al.**

Selection of Study Groups

- Representativeness of the exposed cohort:
  The study included 16,609 women who delivered singleton babies in a tertiary hospital in Spain. Women were categorized by BMI based on WHO standards, focusing on obese (BMI ≥ 30) and normal-weight (BMI 18.5–24.9) groups. This large cohort is highly representative of the population served by the institution but may not generalize to other settings.
  Score: 1 (Somewhat representative).
- Selection of the non-exposed cohort:
  Normal-weight women served as the non-exposed reference group, ensuring clear comparisons with obese women.
  Score: 1.
- Ascertainment of exposure:
  BMI was calculated using self-reported weight and height at the first antenatal visit. Although validated in previous research, self-reported measures may introduce inaccuracies.
  Score: 1.
- Demonstration that outcome of interest was not present at the start of the study:
  Outcomes such as cesarean section, preeclampsia, and neonatal admission to NICU were assessed after delivery, ensuring they were not pre-existing conditions.
  Score: 1.

2. Comparability of Groups

- Comparability based on design or analysis:
  The study adjusted for key confounders, including maternal age, parity, gestational age, and chronic hypertension. However, it did not account for other potential confounders, such as lifestyle factors or socioeconomic status.
  Score: 1 (Adjusted for key confounders).

3. Outcome Assessment

- Assessment of outcomes:
  Maternal and neonatal outcomes (e.g., cesarean delivery, preeclampsia, neonatal intensive care admission) were collected from the Cruces Perinatal Database, which uses standardized clinical records, ensuring reliable outcome measurement.
  Score: 1.
- Follow-up duration:
  The study did not include long-term follow-up of mothers or neonates beyond the immediate postpartum period, limiting the scope of its findings.
  Score: 0 (No long-term follow-up).
- Adequacy of follow-up of cohorts:
  Data completeness was high, with a large sample size and detailed categorization. No evidence of significant loss to follow-up was reported.
  Score: 1.

Total NOS Score: 7/9

- Interpretation:
  This study demonstrates a low risk of bias, with robust methodology for exposure and outcome assessment and adjustments for confounders. The primary limitation is the absence of long-term follow-up.

**Study 17. Oldfield et al.**

**Selection of Study Groups**

- **Representativeness of the exposed cohort:**
  The study included 42 eumenorrheic women aged 19–38 years recruited from the general population. Obesity was defined using total body fat percentage measured by dual-energy X-ray absorptiometry (DEXA). The focus on eumenorrheic women ensures a specific reproductive phenotype, but the population may not represent all women with obesity.
  **Score: 1 (Somewhat representative).**
- **Selection of the non-exposed cohort:**
  Normal-weight women (total body fat < 35%) were included as the non-exposed comparison group, enabling direct comparisons to the obese group.
  **Score: 1.**
- **Ascertainment of exposure:**
  Exposure (obesity) was ascertained using objective, precise DEXA measurements, which are more reliable than BMI alone.
  **Score: 1 (Secure records).**
- **Demonstration that outcome of interest was not present at the start of the study:**
  Outcomes related to antral follicle dynamics were assessed during the study period, ensuring no pre-existing biases in outcomes.
  **Score: 1.**

**2. Comparability of Groups**

- **Comparability based on design or analysis:**
  The study adjusted for potential confounders such as age and cycle phase in analyses of follicle and endocrine data. However, other lifestyle factors (e.g., diet, exercise) were not accounted for, potentially affecting the findings.
  **Score: 1 (Adjusted for key confounders).**

**3. Outcome Assessment**

- **Assessment of outcomes:**
  Antral follicle dynamics were evaluated using transvaginal ultrasonography and well-validated endocrine assays, ensuring reliable outcome measurements.
  **Score: 1.**
- **Follow-up duration:**
  The study was cross-sectional and focused on a single inter-ovulatory interval (IOI), without long-term follow-up on reproductive outcomes.
  **Score: 0 (No long-term follow-up).**
- **Adequacy of follow-up of cohorts:**
  Data completeness was high, with 42 participants completing the study protocol, and no significant missing data reported.
  **Score: 1.**

**Total NOS Score: 7/9**

- **Interpretation:**
  This study demonstrates a low risk of bias, with robust methodology for exposure and outcome assessment and adjustments for key confounders. The primary limitation is the lack of long-term follow-up.

**Study 18. Paasch et al.**

Selection of Study Groups

- Representativeness of the exposed cohort:
  The study included 2,157 men aged 17–67 years who attended infertility clinics in Germany. The cohort was stratified by BMI categories (underweight, normal weight, overweight, and obese). While the sample size is large and diverse, it represents men seeking fertility treatment and may not generalize to all men.
  Score: 1 (Somewhat representative).
- Selection of the non-exposed cohort:
  Normal-weight men (BMI 20–25 kg/m²) served as the control group for comparisons, ensuring a clear reference for exposure (BMI).
  Score: 1.
- Ascertainment of exposure:
  BMI was calculated using measured weight and height, ensuring reliable exposure assessment.
  Score: 1 (Secure records).
- Demonstration that outcome of interest was not present at the start of the study:
  Outcomes such as sperm count, motility, and hormone levels (testosterone, inhibin-B) were assessed during the study, ensuring no pre-existing outcome bias.
  Score: 1.

2. Comparability of Groups

- Comparability based on design or analysis:
  The study controlled for age in the regression models, a significant confounder for fertility parameters. However, other lifestyle or environmental factors (e.g., smoking, physical activity) were not accounted for.
  Score: 1 (Adjusted for key confounders).

3. Outcome Assessment

- Assessment of outcomes:
  Outcomes such as total sperm count, motility, and hormone levels were assessed using standardized laboratory techniques (e.g., CASA for sperm motility, WHO guidelines for semen analysis, and validated assays for hormone measurements).
  Score: 1.
- Follow-up duration:
  This was a cross-sectional study, focusing on a single semen analysis per participant, with no follow-up to assess reproductive outcomes such as live births.
  Score: 0 (No follow-up).
- Adequacy of follow-up of cohorts:
  The study reported data for all participants with no significant exclusions, ensuring data completeness.
  Score: 1.

Total NOS Score: 7/9

- Interpretation:
  This study demonstrates a low risk of bias, with strengths in its exposure and outcome assessments and adjustments for age as a key confounder. However, limitations include its cross-sectional design and lack of adjustment for other confounders like lifestyle factors.

**Study 19: Pinborg et al.**

Selection of Study Groups

- Representativeness of the exposed cohort:
  The study included 487 infertile couples undergoing IVF/ICSI cycles from multiple fertility clinics in Denmark. The cohort represents couples accessing public fertility treatments in Denmark and is reasonably representative of this population.
  Score: 1 (Somewhat representative).
- Selection of the non-exposed cohort:
  Normal-weight women (BMI 18.5–24.9 kg/m²) were used as the reference group for comparison with underweight, overweight, and obese women.
  Score: 1.
- Ascertainment of exposure:
  BMI was calculated using measured height and weight recorded during the initial treatment visit, ensuring reliable exposure assessment.
  Score: 1 (Secure records).
- Demonstration that outcome of interest was not present at the start of the study:
  Outcomes (e.g., live birth, oocyte count, embryo quality) were assessed post-treatment, ensuring they were not pre-existing.
  Score: 1.

2. Comparability of Groups

- Comparability based on design or analysis:
  The study adjusted for key confounders, including age, infertility diagnosis, duration of infertility, and socioeconomic status. However, lifestyle factors such as smoking were not fully accounted for.
  Score: 1 (Adjusted for key confounders).

3. Outcome Assessment

- Assessment of outcomes:
  Outcomes such as live birth, pregnancy, and embryo quality were measured using standardized clinical definitions and protocols. Data collection was systematic across the 5-year follow-up period.
  Score: 1.
- Follow-up duration:
  The study followed couples for up to 5 years and included all IVF/ICSI cycles during this period, providing robust data on cumulative outcomes.
  Score: 1 (Adequate follow-up).
- Adequacy of follow-up of cohorts:
  Only 1.1% of couples were lost to follow-up, and the data set was complete for the primary outcomes.
  Score: 1.

Total NOS Score: 8/9

- Interpretation:
  The study demonstrates a low risk of bias, with strengths in its representative cohort, exposure and outcome measurement, and long-term follow-up. The main limitation is the lack of detailed adjustment for lifestyle confounders.

**Study 20: Raad et al.**

Selection of Study Groups

- Representativeness of the exposed cohort:
  The study included 128 infertile men stratified into lean, overweight, and obese groups. Participants were recruited from a single fertility clinic in Lebanon, focusing on those seeking assisted reproductive treatment. While this cohort is specific to infertile men, it may not represent all men globally.
  Score: 1 (Somewhat representative).
- Selection of the non-exposed cohort:
  Lean men (BMI < 25) were used as the control group, providing a clear comparison to overweight and obese groups.
  Score: 1.
- Ascertainment of exposure:
  BMI and waist circumference were measured objectively, ensuring reliable exposure classification.
  Score: 1 (Secure records).
- Demonstration that outcome of interest was not present at the start of the study:
  Outcomes such as sperm DNA methylation and embryo morphokinetics were measured during the study, ensuring no pre-existing outcomes.
  Score: 1.

2. Comparability of Groups

- Comparability based on design or analysis:
  The study accounted for confounders such as smoking, alcohol consumption, and prior diseases. However, other lifestyle factors (e.g., physical activity) were not controlled for, which could influence sperm quality and embryonic development.
  Score: 1 (Adjusted for some confounders).

3. Outcome Assessment

- Assessment of outcomes:
  Sperm and embryonic outcomes were assessed using validated laboratory techniques and time-lapse imaging, ensuring reliable and reproducible results.
  Score: 1.
- Follow-up duration:
  The study evaluated outcomes during IVF and early embryonic development but did not include follow-up for live birth rates or neonatal health.
  Score: 0 (No long-term follow-up).
- Adequacy of follow-up of cohorts:
  Data completeness was ensured, with no significant exclusions or missing data reported for the primary outcomes.
  Score: 1.

Total NOS Score: 7/9

- Interpretation:
  The study demonstrates a low risk of bias, with strengths in its representative cohort, validated outcome measures, and adjustment for key confounders. Its main limitation is the lack of long-term follow-up for live births or child health outcomes.

**Study 21: Sabolović Rudman et al.**

Selection of Study Groups

- Representativeness of the exposed cohort:
  The study included 51 obese pregnant women and 50 normal-BMI pregnant women, all diagnosed with gestational diabetes mellitus (GDM) according to IADPSG criteria. The cohort is representative of pregnant women with GDM but may not generalize to the broader population of pregnant women without GDM.
  Score: 1 (Somewhat representative).
- Selection of the non-exposed cohort:
  Pregnant women with normal BMI served as the non-exposed reference group, enabling direct comparisons with obese women.
  Score: 1.
- Ascertainment of exposure:
  BMI was calculated based on measured height and weight during pregnancy, ensuring reliable exposure assessment.
  Score: 1 (Secure records).
- Demonstration that outcome of interest was not present at the start of the study:
  Outcomes such as gestational weight gain, neonatal birth weight, and hypertrophic newborns were assessed during or after pregnancy, ensuring they were not pre-existing.
  Score: 1.

2. Comparability of Groups

- Comparability based on design or analysis:
  The study accounted for gestational diabetes in both groups, reducing a major potential confounder. However, it did not adjust for other relevant confounders, such as maternal age, parity, or lifestyle factors.
  Score: 1 (Partially adjusted for confounders).

3. Outcome Assessment

- Assessment of outcomes:
  Outcomes such as gestational weight gain, hypertension, neonatal birth weight, and neonatal hypertrophy were measured using clinical records and standardized protocols.
  Score: 1.
- Follow-up duration:
  The study did not follow participants beyond delivery to assess long-term maternal or neonatal outcomes, such as postpartum weight retention or neonatal growth.
  Score: 0 (No long-term follow-up).
- Adequacy of follow-up of cohorts:
  Data completeness appears adequate for the analyzed outcomes, with no significant exclusions reported.
  Score: 1.

Total NOS Score: 7/9

- Interpretation:
  The study demonstrates a low risk of bias, with strengths in exposure and outcome assessment and a well-defined control group. The primary limitations are the lack of adjustment for additional confounders and the absence of long-term follow-up.

**Study 22. Shen et al.**

Selection of Study Groups

- Representativeness of the exposed cohort:
  The study included 2,292 pregnant women registered in Shanghai, categorized by BMI (underweight, normal weight, overweight, and obese). The cohort represents pregnant women in Shanghai who attended a specialized healthcare hospital, but its findings may not generalize to rural or less urbanized populations.
  Score: 1 (Somewhat representative).
- Selection of the non-exposed cohort:
  Normal-weight pregnant women (BMI 18.5–23.9) were used as the reference group, allowing for direct comparisons with overweight and obese women.
  Score: 1.
- Ascertainment of exposure:
  Prepregnancy BMI was calculated using measured weight and height at the first antenatal visit, ensuring objective and reliable exposure assessment.
  Score: 1 (Secure records).
- Demonstration that outcome of interest was not present at the start of the study:
  Outcomes such as gestational weight gain, delivery mode, and maternal/neonatal complications were assessed post-pregnancy, ensuring they were not pre-existing.
  Score: 1.

2. Comparability of Groups

- Comparability based on design or analysis:
  The study adjusted for confounders such as maternal age, parity, and gravidity in multivariate analyses. However, lifestyle factors like diet and physical activity, which may influence pregnancy outcomes, were not accounted for.
  Score: 1 (Adjusted for some confounders).

3. Outcome Assessment

- Assessment of outcomes:
  Pregnancy outcomes, including gestational weight gain, cesarean delivery, macrosomia, and maternal complications, were assessed using clinical records and validated diagnostic criteria, ensuring reliability.
  Score: 1.
- Follow-up duration:
  The study followed participants through delivery but did not assess long-term maternal or neonatal outcomes, such as postpartum weight retention or neonatal health.
  Score: 0 (No long-term follow-up).
- Adequacy of follow-up of cohorts:
  Data completeness was high, with detailed records for 2,292 women. Loss to follow-up occurred primarily due to participants delivering at other hospitals, but exclusions were well-documented.
  Score: 1.

Total NOS Score: 7/9

- Interpretation:
  This study demonstrates a low risk of bias, with strengths in exposure and outcome measurement and adjustments for key confounders. Limitations include the absence of long-term follow-up and unadjusted lifestyle confounders.

**Study 23. Aly et al.**

Selection of Study Groups

- Representativeness of the exposed cohort:
  The study included 14,183 singleton pregnancies from the George Washington University Medical Center between 1992 and 2003, stratified into non-obese, obese, and morbidly obese groups. The cohort is reasonably representative of urban pregnant women receiving care in the U.S., but it may not generalize to rural or underserved populations.
  Score: 1 (Somewhat representative).
- Selection of the non-exposed cohort:
  Non-obese women (BMI < 30) were used as the reference group, ensuring a clear comparison with obese and morbidly obese groups.
  Score: 1.
- Ascertainment of exposure:
  BMI was calculated using measured weight and height recorded at the first prenatal visit, ensuring objective and accurate exposure classification.
  Score: 1 (Secure records).
- Demonstration that outcome of interest was not present at the start of the study:
  Outcomes such as prematurity and low birth weight (LBW) were assessed during or after delivery, ensuring these were not pre-existing.
  Score: 1.

2. Comparability of Groups

- Comparability based on design or analysis:
  The study controlled for confounders such as maternal age, gravidity, smoking, anemia, hypertension, and diabetes using logistic regression models. However, additional lifestyle factors (e.g., physical activity or socioeconomic status) were not included.
  Score: 1 (Adjusted for key confounders).

3. Outcome Assessment

- Assessment of outcomes:
  Outcomes, including prematurity (<37 weeks), LBW, and maternal complications (e.g., diabetes, hypertension), were assessed using validated clinical records, ensuring reliability.
  Score: 1.
- Follow-up duration:
  The study did not include follow-up beyond delivery, omitting long-term maternal or neonatal outcomes.
  Score: 0 (No long-term follow-up).
- Adequacy of follow-up of cohorts:
  Data completeness was ensured for the large cohort, with detailed statistical analysis minimizing bias. Excluded cases were well-documented and did not appear to bias the results.
  Score: 1.

Total NOS Score: 7/9

- Interpretation:
  The study demonstrates a low risk of bias, with strengths in exposure and outcome measurement, adjustment for key confounders, and the use of a large dataset. The primary limitation is the lack of long-term follow-up for maternal or neonatal outcomes.

**Study 24. Bhandari et al.**

Selection of Study Groups

- Representativeness of the exposed cohort:
  The study included 414 women with recurrent miscarriage (RMC) attending a tertiary implantation clinic in the UK. While this cohort represents women with recurrent pregnancy losses referred to specialized care, it may not generalize to women experiencing RMC in non-tertiary settings.
  Score: 1 (Somewhat representative).
- Selection of the non-exposed cohort:
  Women were grouped by BMI categories (normal weight, overweight, and obese) with normal-weight women as the reference. However, the study lacked a broader non-exposed group (e.g., women without RMC).
  Score: 0.
- Ascertainment of exposure:
  BMI was calculated using measured height and weight at the time of consultation, ensuring reliable exposure assessment.
  Score: 1 (Secure records).
- Demonstration that outcome of interest was not present at the start of the study:
  Outcomes such as superfertility and pregnancy loss patterns were assessed retrospectively but were not pre-existing when BMI was recorded.
  Score: 1.

2. Comparability of Groups

- Comparability based on design or analysis:
  The study adjusted for maternal age across BMI categories. However, other confounders such as lifestyle factors, parity, and comorbidities were not accounted for, which may have influenced outcomes.
  Score: 1 (Partial adjustment for confounders).

3. Outcome Assessment

- Assessment of outcomes:
  Pregnancy loss patterns and time-to-pregnancy (TTP) were assessed using detailed clinical records, including validated diagnostic criteria for miscarriage subtypes.
  Score: 1.
- Follow-up duration:
  The study assessed pregnancy outcomes retrospectively, focusing on historical pregnancies rather than prospective follow-up of current or future pregnancies.
  Score: 0 (No long-term follow-up).
- Adequacy of follow-up of cohorts:
  Data completeness appears high, with all 414 participants contributing to the final analyses. Recall bias is acknowledged but was mitigated by using structured clinical interviews and medical records.
  Score: 1.

Total NOS Score: 6/9

- Interpretation:
  The study demonstrates moderate risk of bias, with strengths in exposure and outcome measurement and adequate cohort size. Limitations include the retrospective nature, lack of a broader non-exposed control group, and limited adjustment for confounders.

**Study 25. Caillon et al.**

**Selection of Study Groups**

- **Representativeness of the exposed cohort:**
  The study included 558 women undergoing IVF or ICSI cycles at a single tertiary center in France, categorized by BMI. While the cohort represents women undergoing fertility treatment, it may not generalize to all infertile women globally.
  **Score: 1 (Somewhat representative).**
- **Selection of the non-exposed cohort:**
  Normal-weight women (BMI 18.5–24.9) served as the non-exposed group, providing a clear comparison with overweight and obese women.
  **Score: 1.**
- **Ascertainment of exposure:**
  BMI was calculated using measured weight and height during infertility workup, ensuring accurate and reliable exposure assessment.
  **Score: 1 (Secure records).**
- **Demonstration that outcome of interest was not present at the start of the study:**
  Outcomes such as ovarian response, embryo quality, and clinical pregnancy were assessed post-treatment, ensuring they were not pre-existing.
  **Score: 1.**

**2. Comparability of Groups**

- **Comparability based on design or analysis:**
  The study adjusted for relevant baseline characteristics such as age, ovarian reserve markers (AMH, AFC), and smoking status. However, other potential confounders like lifestyle factors and male factors were not explicitly accounted for.
  **Score: 1 (Adjusted for key confounders).**

**3. Outcome Assessment**

- **Assessment of outcomes:**
  Outcomes such as ovarian stimulation results, embryo quality, and pregnancy rates were assessed using standardized clinical criteria and validated laboratory protocols.
  **Score: 1.**
- **Follow-up duration:**
  The study assessed only short-term outcomes, such as implantation and clinical pregnancy rates, without follow-up on live birth rates or long-term neonatal outcomes.
  **Score: 0 (No long-term follow-up).**
- **Adequacy of follow-up of cohorts:**
  Data completeness was ensured for all participants, with no significant exclusions or missing data for primary outcomes.
  **Score: 1.**

**Total NOS Score: 7/9**

- **Interpretation:**
  This study demonstrates a low risk of bias, with strengths in its rigorous methodology for exposure and outcome measurement, as well as adjustment for key confounders. The primary limitation is the absence of long-term follow-up.

**Study 26. Grieger et al.**

Selection of Study Groups

- Representativeness of the exposed cohort:
  The study included 5,519 nulliparous women from multiple international centers (Australia, Ireland, New Zealand, and the UK) who were assessed for metabolic syndrome (MetS) and its impact on time to pregnancy (TTP) and infertility. While the sample represents a diverse population, it may not generalize to all nulliparous women worldwide.
  Score: 1 (Somewhat representative).
- Selection of the non-exposed cohort:
  Women without MetS served as the non-exposed group, allowing direct comparisons.
  Score: 1.
- Ascertainment of exposure:
  MetS was assessed using the International Diabetes Federation criteria, incorporating objective measurements of waist circumference, triglycerides, high-density lipoprotein (HDL), blood pressure, and blood glucose.
  Score: 1 (Secure records).
- Demonstration that outcome of interest was not present at the start of the study:
  Outcomes such as TTP and infertility were measured retrospectively and only included participants who achieved conception, ensuring that outcomes were not pre-existing at the study's start.
  Score: 1.

2. Comparability of Groups

- Comparability based on design or analysis:
  Adjustments were made for confounders such as maternal age, BMI, socio-economic index, ethnicity, and paternal BMI, providing a robust analysis. However, lifestyle factors like physical activity were not considered.
  Score: 1 (Adjusted for key confounders).

3. Outcome Assessment

- Assessment of outcomes:
  TTP and infertility were measured retrospectively using a well-defined questionnaire and clinical records. While retrospective TTP can introduce recall bias, this was mitigated through structured data collection.
  Score: 1.
- Follow-up duration:
  The study was cross-sectional and did not include follow-up to assess long-term fertility outcomes or subsequent pregnancies.
  Score: 0 (No long-term follow-up).
- Adequacy of follow-up of cohorts:
  The study reported complete data for 5,519 women, with detailed analysis and sensitivity checks for missing or incomplete data.
  Score: 1.

Total NOS Score: 7/9

- Interpretation:
  The study demonstrates a low risk of bias, with strengths in its diverse sample population, validated exposure and outcome assessments, and adjustment for confounders. The primary limitation is the lack of long-term follow-up.

**Study 27. Haghighi et al.**

Selection of Study Groups

- Representativeness of the exposed cohort:
  The study included 230 women undergoing their first IVF cycle at a single hospital in Iran, stratified into BMI categories. While the cohort reflects women in a hospital-based fertility treatment setting, its findings may not generalize to all women undergoing IVF globally.
  Score: 1 (Somewhat representative).
- Selection of the non-exposed cohort:
  Normal-weight women (BMI 20–27.9 kg/m²) served as the reference group, enabling comparisons with underweight (<20 kg/m²) and obese (>28 kg/m²) women.
  Score: 1.
- Ascertainment of exposure:
  BMI was calculated based on measured weight and height, ensuring a reliable and accurate exposure assessment.
  Score: 1 (Secure records).
- Demonstration that outcome of interest was not present at the start of the study:
  IVF outcomes, including clinical pregnancy and embryo transfer success, were measured after intervention, ensuring outcomes were not pre-existing.
  Score: 1.

2. Comparability of Groups

- Comparability based on design or analysis:
  The study grouped participants by BMI and adjusted for basic confounders like age and infertility duration. However, lifestyle factors and male partner characteristics were not considered.
  Score: 1 (Partial adjustment for confounders).

3. Outcome Assessment

- Assessment of outcomes:
  IVF outcomes, including endometrial thickness, embryo transfer success, and pregnancy rates, were assessed using standardized clinical protocols. The clinical pregnancy definition included the presence of a gestational sac on ultrasound.
  Score: 1.
- Follow-up duration:
  The study only evaluated immediate IVF outcomes, such as clinical pregnancy, without long-term follow-up on live births or neonatal health.
  Score: 0 (No long-term follow-up).
- Adequacy of follow-up of cohorts:
  The study appears to have complete data for all participants, with no major exclusions or missing data reported.
  Score: 1.

Total NOS Score: 7/9

- Interpretation:
  The study demonstrates a low risk of bias, with robust exposure and outcome measurements and partial adjustment for confounders. The primary limitation is the absence of long-term follow-up and broader adjustments for potential confounders.

**Study 28. Joy et al.**

Selection of Study Groups

- Representativeness of the exposed cohort:
  The study included 12,915 women with singleton pregnancies delivering at term, stratified by BMI. These women were from a high-risk pregnancy database in the U.S., which makes the cohort representative of women receiving specialized perinatal care but may not fully generalize to all pregnant women.
  Score: 1 (Somewhat representative).
- Selection of the non-exposed cohort:
  Normal-weight women (BMI 20–24.9 kg/m²) were used as the reference group, ensuring a clear comparison with obese women (BMI ≥ 30).
  Score: 1.
- Ascertainment of exposure:
  BMI was calculated using documented prepregnancy weight and height, ensuring reliable and accurate exposure classification.
  Score: 1 (Secure records).
- Demonstration that outcome of interest was not present at the start of the study:
  Outcomes such as gestational diabetes, gestational hypertension, and delivery mode were assessed after exposure, ensuring they were not pre-existing.
  Score: 1.

2. Comparability of Groups

- Comparability based on design or analysis:
  The study adjusted for key confounders such as maternal age, race, and parity in statistical analyses. However, other lifestyle or behavioral factors, like smoking or physical activity, were not included.
  Score: 1 (Partial adjustment for confounders).

3. Outcome Assessment

- Assessment of outcomes:
  Maternal and neonatal outcomes (e.g., gestational diabetes, gestational hypertension, birth weight, large-for-gestational-age infants) were assessed using standardized clinical criteria and medical records, ensuring reliable outcome measurement.
  Score: 1.
- Follow-up duration:
  The study did not include follow-up beyond delivery, omitting long-term maternal and neonatal health outcomes.
  Score: 0 (No long-term follow-up).
- Adequacy of follow-up of cohorts:
  Data completeness was high, with detailed records for 12,915 women and minimal exclusions.
  Score: 1.

Total NOS Score: 7/9

- Interpretation:
  This study demonstrates a low risk of bias, with robust methodologies for exposure and outcome measurement and adjustment for key confounders. The main limitation is the lack of long-term follow-up.

**Study 29. Lin et al.**

Selection of Study Groups

- Representativeness of the exposed cohort:
  The study included 189 infertile women undergoing IVF, with 125 classified as normal BMI and 64 as overweight/obese. The cohort was drawn from a single reproductive center in China, which is representative of women undergoing fertility treatment in a specialized setting but may not generalize to other populations.
  Score: 1 (Somewhat representative).
- Selection of the non-exposed cohort:
  Women with normal BMI (18.5–23.9) served as the non-exposed group, enabling a clear comparison with overweight/obese women.
  Score: 1.
- Ascertainment of exposure:
  BMI was calculated using measured weight and height during the IVF process, ensuring reliable exposure classification.
  Score: 1 (Secure records).
- Demonstration that outcome of interest was not present at the start of the study:
  Outcomes such as embryo quality and clinical pregnancy were assessed after IVF treatment, ensuring they were not pre-existing.
  Score: 1.

2. Comparability of Groups

- Comparability based on design or analysis:
  The study adjusted for baseline variables such as age, gonadotropin dose, and ovarian response. However, other confounders such as lifestyle and male partner characteristics were not included.
  Score: 1 (Partial adjustment for confounders).

3. Outcome Assessment

- Assessment of outcomes:
  IVF outcomes, including good-quality embryo rate, clinical pregnancy, and live birth, were assessed using standard clinical definitions. Laboratory protocols for embryo assessment were consistent and validated.
  Score: 1.
- Follow-up duration:
  The study reported immediate IVF outcomes, such as live birth rates, but did not follow participants or offspring for long-term health outcomes.
  Score: 0 (No long-term follow-up).
- Adequacy of follow-up of cohorts:
  The study maintained complete data for all 189 participants, with no significant exclusions reported.
  Score: 1.

Total NOS Score: 7/9

- Interpretation:
  This study demonstrates a low risk of bias, with strengths in exposure and outcome assessment and partial confounder adjustment. The main limitations include the lack of adjustment for additional confounders and the absence of long-term follow-up.

**Study 30. Orvieto et al.**

Selection of Study Groups

- Representativeness of the exposed cohort:
  The study included 189 women undergoing ovarian stimulation (OS) for IVF at a single tertiary medical center, categorized into obese (BMI ≥ 30) and non-obese (BMI < 30) groups. The cohort represents women undergoing IVF in a specialized setting but may not generalize to all infertile women globally.
  Score: 1 (Somewhat representative).
- Selection of the non-exposed cohort:
  Non-obese women (BMI < 30) served as the reference group, providing a clear comparison to the obese group.
  Score: 1.
- Ascertainment of exposure:
  BMI was calculated based on measured height and weight during the study, ensuring accurate and reliable exposure assessment.
  Score: 1 (Secure records).
- Demonstration that outcome of interest was not present at the start of the study:
  Outcomes, including oocyte and embryo quality, were assessed after ovarian stimulation and retrieval, ensuring they were not pre-existing.
  Score: 1.

2. Comparability of Groups

- Comparability based on design or analysis:
  The study did not adjust for confounding factors such as age, ovarian reserve, or gonadotropin dose differences, despite these being potential confounders.
  Score: 0.

3. Outcome Assessment

- Assessment of outcomes:
  Outcomes such as oocyte recovery, fertilization rate, and top-quality embryo (TQE) rates were measured using standardized laboratory and clinical protocols, ensuring reliability.
  Score: 1.
- Follow-up duration:
  The study did not assess live birth rates or neonatal outcomes, focusing only on immediate IVF outcomes such as oocyte and embryo quality.
  Score: 0 (No long-term follow-up).
- Adequacy of follow-up of cohorts:
  The study included all 189 participants, with no significant exclusions or missing data reported.
  Score: 1.

Total NOS Score: 6/9

- Interpretation:
  The study demonstrates moderate risk of bias, with strengths in exposure and outcome measurement and adequate follow-up. However, the lack of adjustment for confounders and absence of long-term follow-up are limitations.

**Study 31: Shulman et al**.

Selection of Study Groups

- Representativeness of the exposed cohort:
  The study included data from 45,163 women undergoing ART embryo transfers from the Society for Assisted Reproductive Technology Clinic Online Reporting System (SART CORS). This cohort is representative of women undergoing ART in the U.S., although it may not fully generalize to non-U.S. populations.
  Score: 1 (Somewhat representative).
- Selection of the non-exposed cohort:
  Women with normal BMI (18.5–24.9) served as the non-exposed comparison group for those classified as overweight or obese.
  Score: 1.
- Ascertainment of exposure:
  BMI was calculated using recorded height and weight, ensuring a reliable and objective exposure classification.
  Score: 1 (Secure records).
- Demonstration that outcome of interest was not present at the start of the study:
  Outcomes such as failure to achieve pregnancy or live birth were assessed during or after ART, ensuring they were not present at baseline.
  Score: 1.

2. Comparability of Groups

- Comparability based on design or analysis:
  The study controlled for key confounders, including age, oocyte source, race, embryo transfer day, and infertility diagnosis. However, additional factors such as lifestyle or comorbidities were not considered.
  Score: 1 (Adjusted for key confounders).

3. Outcome Assessment

- Assessment of outcomes:
  Outcomes (e.g., clinical pregnancy, live birth) were assessed using standardized clinical definitions and recorded in a national database, ensuring reliable outcome measurement.
  Score: 1.
- Follow-up duration:
  The study assessed outcomes up to live birth but did not evaluate long-term maternal or neonatal health outcomes.
  Score: 0 (No long-term follow-up).
- Adequacy of follow-up of cohorts:
  Data completeness appears high, given the use of the SART CORS database. Missing data and exclusions were minimal and well-documented.
  Score: 1.

Total NOS Score: 7/9

- Interpretation:
  This study demonstrates a low risk of bias, with robust exposure and outcome assessment and appropriate adjustment for confounders. The primary limitation is the absence of long-term follow-up.

**Study 32: Veleva et al.**

Selection of Study Groups

- Representativeness of the exposed cohort:
  The study analyzed 3,330 first clinical pregnancy cycles from a large cohort undergoing assisted reproductive technologies (ART) in Finland. The population represents women undergoing ART treatments and reflects a specialized clinical setting.
  Score: 1 (Somewhat representative).
- Selection of the non-exposed cohort:
  Women with normal BMI (18.5–24.9 kg/m²) served as the reference group, allowing comparisons with underweight and obese groups.
  Score: 1.
- Ascertainment of exposure:
  BMI was calculated using measured height and weight during treatment, ensuring a reliable and objective assessment of exposure.
  Score: 1 (Secure records).
- Demonstration that outcome of interest was not present at the start of the study:
  Miscarriage outcomes were assessed after ART treatments, ensuring that outcomes were not present at the study’s start.
  Score: 1.

2. Comparability of Groups

- Comparability based on design or analysis:
  The study adjusted for key confounders, including age, type of infertility, previous miscarriage history, and ART cycle type. However, potential lifestyle factors or male partner characteristics were not considered.
  Score: 1 (Partial adjustment for confounders).

3. Outcome Assessment

- Assessment of outcomes:
  Miscarriage rates were defined and measured using clinical pregnancy criteria, including transvaginal ultrasonography for gestational sac and cardiac activity.
  Score: 1.
- Follow-up duration:
  The study assessed miscarriage rates up to 12 weeks of gestation but did not include live birth rates or longer-term follow-up.
  Score: 0 (No long-term follow-up).
- Adequacy of follow-up of cohorts:
  Data were complete for the 3,330 cycles analyzed, with a robust approach to missing data management.
  Score: 1.

Total NOS Score: 7/9

- Interpretation:
  This study demonstrates a low risk of bias, with strengths in its exposure and outcome measurement, large sample size, and confounder adjustments. The primary limitation is the absence of long-term follow-up for live birth or neonatal outcomes.

**Study 33: Yogev and Langer**

**Selection of Study Groups**

- **Representativeness of the exposed cohort:**
  The study included 1,319 obese and morbidly obese women with gestational diabetes mellitus (GDM) treated at a single medical center in the U.S. While the population reflects a specialized clinical setting, it may not generalize to women with GDM outside of tertiary care.
  **Score: 1 (Somewhat representative).**
- **Selection of the non-exposed cohort:**
  The study categorized patients into obese (BMI 30–34.9) and morbidly obese (BMI ≥ 35) groups but did not include a non-obese comparison group.
  **Score: 0.**
- **Ascertainment of exposure:**
  BMI was calculated based on measured prepregnancy weight and height recorded in clinical charts, ensuring a reliable and objective classification of exposure.
  **Score: 1 (Secure records).**
- **Demonstration that outcome of interest was not present at the start of the study:**
  Outcomes such as cesarean delivery, neonatal macrosomia, and metabolic complications were assessed during or after delivery, ensuring they were not present at baseline.
  **Score: 1.**

**2. Comparability of Groups**

- **Comparability based on design or analysis:**
  The study adjusted for some confounders, such as glycemic control and treatment modality (diet vs. insulin), but did not control for other factors like age, parity, or socioeconomic status that could influence outcomes.
  **Score: 1 (Partial adjustment for confounders).**

**3. Outcome Assessment**

- **Assessment of outcomes:**
  Pregnancy outcomes, including large-for-gestational-age (LGA), neonatal complications, and cesarean rates, were assessed using standardized clinical protocols and medical records.
  **Score: 1.**
- **Follow-up duration:**
  The study did not evaluate long-term maternal or neonatal outcomes, focusing only on outcomes up to delivery.
  **Score: 0 (No long-term follow-up).**
- **Adequacy of follow-up of cohorts:**
  Data completeness was high, with no major exclusions or missing data for the primary outcomes.
  **Score: 1.**

**Total NOS Score: 6/9**

- **Interpretation:**
  This study demonstrates moderate risk of bias, with strengths in exposure and outcome measurement and partial adjustment for confounders. Its main limitations include the absence of a non-obese comparison group and long-term follow-up.

**Study 34: Zhang et al.**

**Selection of Study Groups**

- **Representativeness of the exposed cohort:**
  The study included 439 women undergoing either conventional IVF (219 participants) or minimal stimulation IVF (220 participants), stratified by BMI into normal, overweight, and obese groups. While this cohort reflects women undergoing ART in a clinical setting, it may not generalize to women outside of specialized fertility clinics.
  **Score: 1 (Somewhat representative).**
- **Selection of the non-exposed cohort:**
  Normal BMI women (BMI 18.5–24.9) served as the reference group, enabling direct comparisons with overweight and obese women.
  **Score: 1.**
- **Ascertainment of exposure:**
  BMI was calculated using measured weight and height at the initial visit, ensuring reliable and objective exposure classification.
  **Score: 1 (Secure records).**
- **Demonstration that outcome of interest was not present at the start of the study:**
  IVF outcomes such as oocyte retrieval, embryo quality, and live birth were assessed after ovarian stimulation, ensuring that outcomes were not pre-existing.
  **Score: 1.**

**2. Comparability of Groups**

- **Comparability based on design or analysis:**
  The study adjusted for key confounders, including age, day 3 FSH, total gonadotropin dose, and days of stimulation, providing a robust analysis.
  **Score: 1 (Adjusted for key confounders).**

**3. Outcome Assessment**

- **Assessment of outcomes:**
  Outcomes such as the number of metaphase II (MII) oocytes, fertilized oocytes, cleavage-stage embryos, clinical pregnancy, and live birth rates were measured using validated clinical definitions and protocols.
  **Score: 1.**
- **Follow-up duration:**
  The study focused on immediate ART outcomes, such as clinical pregnancy and live birth rates, without assessing long-term maternal or neonatal health.
  **Score: 0 (No long-term follow-up).**
- **Adequacy of follow-up of cohorts:**
  Data completeness appears adequate, with detailed records for all participants and no evidence of major exclusions or missing data.
  **Score: 1.**

**Total NOS Score: 7/9**

- **Interpretation:**
  This study demonstrates a low risk of bias, with strengths in its rigorous exposure and outcome assessments and adjustments for confounders. The primary limitation is the lack of long-term follow-up.

**Study 35: He et al.**

**Risk of Bias Assessment (RoB 2 Tool)**

**1. Bias Arising from the Randomization Process**

- **Random sequence generation:**
  The study mentions that it is a multicenter RCT with proper randomization of participants into treatment groups. However, details about the method of randomization (e.g., computer-generated random sequence) were not provided in the secondary analysis.
  **Risk: Low (assuming proper randomization in the original trial).**
- **Allocation concealment:**
  The allocation procedure is not detailed in the secondary analysis, but as a large multicenter trial, it likely followed standard allocation concealment procedures.
  **Risk: Low.**
- **Baseline imbalances:**
  No significant imbalances were reported between groups at baseline, except for factors expected to vary with MetS status (e.g., BMI, HOMA-IR).
  **Risk: Low.**

**2. Bias Due to Deviations from Intended Interventions**

- **Blinding of participants and personnel:**
  Participants and clinicians were likely not blinded, as this is a trial involving IVF treatment, where blinding is inherently challenging. However, blinding is less critical for objective IVF outcomes (e.g., live birth rates).
  **Risk: Low.**
- **Adherence to intervention:**
  The study followed standardized ovarian stimulation protocols for all participants, with adjustments based on clinical responses. No major deviations were reported.
  **Risk: Low.**

**3. Bias Due to Missing Outcome Data**

- **Completeness of data:**
  The trial analyzed cumulative live birth rates for 1,508 participants. Missing data were not reported, and exclusions appear minimal and unrelated to outcomes.
  **Risk: Low.**

**4. Bias in Measurement of Outcomes**

- **Blinding of outcome assessors:**
  Outcome measurements (e.g., live birth rates, embryo quality) were based on objective clinical data, reducing the risk of measurement bias.
  **Risk: Low.**
- **Reliability of outcome measurement:**
  Outcomes like cumulative live birth rates were clearly defined and measured according to clinical standards.
  **Risk: Low.**

**5. Bias in Selection of Reported Results**

- **Outcome reporting:**
  All relevant outcomes, including cumulative live birth rates, were reported. The secondary analysis does not appear to have selectively reported outcomes.
  **Risk: Low.**

**Overall Risk of Bias: Low**

The study appears to have low risk of bias across all domains, with appropriate randomization, objective outcome measurement, and minimal missing data. The primary limitation is the lack of detailed reporting on allocation concealment and blinding procedures in the secondary analysis.
